# Immunosuppression broadens evolutionary pathways to drug resistance and treatment failure during *Acinetobacter baumannii* pneumonia in mice

**DOI:** 10.1038/s41564-022-01126-8

**Published:** 2022-05-26

**Authors:** Wenwen Huo, Lindsay M. Busch, Juan Hernandez-Bird, Efrat Hamami, Christopher W. Marshall, Edward Geisinger, Vaughn S. Cooper, Tim van Opijnen, Jason W. Rosch, Ralph R. Isberg

**Affiliations:** 1 Department of Molecular Biology and Microbiology, Tufts University School of Medicine, Boston, MA USA; 2 Department of Microbiology and Molecular Genetics and Center for Evolutionary Biology and Medicine, University of Pittsburgh School of Medicine, Pittsburgh, PA USA; 3 Department of Biology, Northeastern University, Boston, MA USA; 4 Department of Biology, Boston College, Boston, MA USA; 5 Department of Infectious Diseases, St Jude Children’s Research Hospital, Memphis, TN USA; 6 Present Address: Division of Infectious Diseases, Emory University School of Medicine, Atlanta, GA USA; 7 Present Address: Department of Biological Sciences, Marquette University, Milwaukee, WI USA

**Keywords:** Antimicrobial resistance, Infection

## Abstract

*Acinetobacter baumannii* is increasingly refractory to antibiotic treatment in healthcare settings. As is true of most human pathogens, the genetic path to antimicrobial resistance (AMR) and the role that the immune system plays in modulating AMR during disease are poorly understood. Here we reproduced several routes to fluoroquinolone resistance, performing evolution experiments using sequential lung infections in mice that are replete with or depleted of neutrophils, providing two key insights into the evolution of drug resistance. First, neutropenic hosts acted as reservoirs for the accumulation of drug resistance during drug treatment. Selection for variants with altered drug sensitivity profiles arose readily in the absence of neutrophils, while immunocompetent animals restricted the appearance of these variants. Secondly, antibiotic treatment failure in the immunocompromised host was shown to occur without clinically defined resistance, an unexpected result that provides a model for how antibiotic failure occurs clinically in the absence of AMR. The genetic mechanism underlying both these results is initiated by mutations activating the drug egress pump regulator AdeL, which drives persistence in the presence of antibiotic. Therefore, antibiotic persistence mutations present a two-pronged risk during disease, causing drug treatment failure in the immunocompromised host while simultaneously increasing the emergence of high-level AMR.

## Abstract (web summary)

Evolution experiments using sequential *Acinetobacter baumannii* lung infections in immunocompetent and immunocompromised mice treated with ciprofloxacin reveal that neutrophil-depleted hosts enable the outgrowth of drug persisters and serve as reservoirs for antibiotic resistance variants.

## Main

*Acinetobacter baumannii* is a Gram-negative opportunistic pathogen, one of the high-priority ESKAPE (*Enterococcus faecium*, *Staphylococcus aureus*, *Klebsiella pneumoniae*, *Acinetobacter baumannii*, *Pseudomonas aeruginosa*, and *Enterobacter spp.*) organisms that are increasingly difficult to treat due to multiple antibiotic resistance^1^. A substantial proportion of healthcare-associated diseases caused by this group of pathogens, such as ventilator-associated pneumonia, is linked to their documented multidrug resistance (MDR)^2–5^. Of particular importance are patients in intensive care units who are critically ill and have depressed immunological clearance mechanisms that increase the risk of infection by MDR pathogens^6,7^. Consequently, the healthcare environment with its immunologically compromised patients could provide a unique niche for selection of MDR isolates^8^. Overlaying these issues is the fact that for many patients in healthcare settings, antibiotic treatment failure is common but is often unexplained, as resistant organisms cannot be identified^9^.

*A. baumannii* clinical isolates have demonstrated a remarkable ability to successfully battle antibiotic treatment in the clinic due to high intrinsic resistance to antimicrobials and the acquisition of drug resistance elements by the organism^10–13^. A critical missing link is a detailed roadmap for the stepwise evolution of antibiotic resistance in the clinic, particularly in identifying *A. baumannii* subpopulations most likely to give rise to drug treatment failure. Furthermore, it is largely unknown whether there exists a patient group that provides the reservoir for antimicrobial resistance (AMR) acquisition. Particularly for healthcare-associated diseases, patient groups susceptible to *A. baumannii* are by their nature compromised in several ways, with the potential for providing reservoirs for AMR evolution. The range of individuals with altered immune function in these settings may allow for a diversity of host targets that can act as primary amplifiers of resistance, with eventual spread to individuals with different sets of susceptibilities. Therefore, as a model for healthcare-associated pneumonia, we aimed to explore whether depletion of a single arm of innate immunity in mice could help shape the antibiotic treatment outcome and support the evolution of resistant organisms.

Fluoroquinolones (FQ) such as ciprofloxacin initially showed excellent activity against *A. baumannii* infections^14^. Members of this drug class inhibit bacterial cell growth by covalently linking to DNA gyrase (*gyrA*) and topoisomerase IV (*parC*), leading to double stranded DNA breaks and cell death^15^. Drug-resistant mutants arise fairly frequently in the clinic, with over 80% of clinical isolates of *A. baumannii* currently being FQ resistant^16^. The most commonly reported mechanisms of ciprofloxacin resistance in the clinic are target protein alterations and the overexpression of efflux pumps. In *A. baumannii*, alterations in target proteins usually evolve in a stepwise fashion, starting with *gyrA* (usually Ser81Leu) followed by *parC* (usually Ser83Leu). Interestingly, in addition to the patient’s underlying condition and hospitalization status, previous exposure to fluoroquinolones is also a risk factor for *A. baumannii* colonization and infection^17^, indicating that resistance to this antibiotic class is linked to either increased pathogenic potential of the isolate or acquisition of MDR. It is unknown whether there exist early adaptive mutations that enable precursor populations of *A. baumannii* to act as ancestors of drug resistance.

There has been limited study of whether AMR can be suppressed by the immune response. Landmark mathematical modelling work argues that the immune response can largely limit the outgrowth of persisters or other bacterial variants that exhibit intermediate resistance levels^18^. Another study indicates that the cytokine response may control waves of AMR variants^19^. Given the limited analysis of how antibiotic resistance evolves in the clinic, and the lack of a detailed interrogation of the role played by the immune response in controlling selection of AMR, we sought to identify the steps that lead to resistance in the presence or absence of a single arm of innate immunity. The rationale behind this approach is that clinical antibiotic resistance is associated with mutations located outside well-characterized drug targets, and these mutations are difficult to identify bioinformatically or predict on the basis of culture studies^20–22^. This approach provides evidence that absence of neutrophil function allows outgrowth of drug persisters and the appearance of fluoroquinolone resistance. In so doing, we identified mutational pathways to drug resistance, with the results tied to the problem of unexplained antibiotic treatment failure.

## Results

### Recapitulation of *A. baumannii* evolved drug resistance

**Fig. 1:**
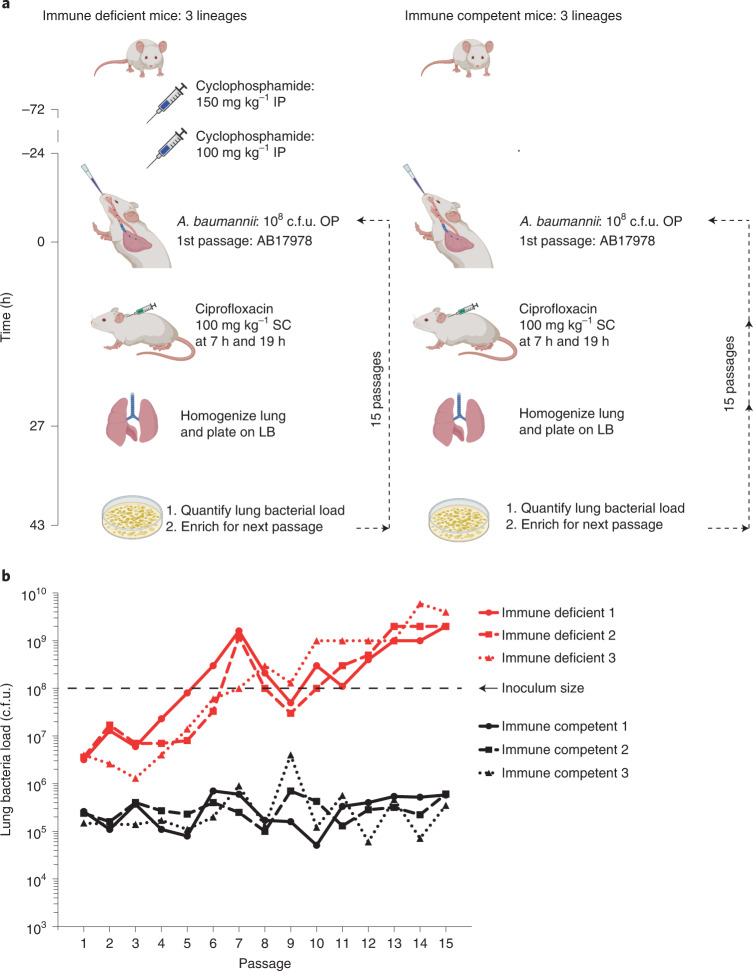
Continuous passaging of *A. baumannii* in a lung infection model results in early antibiotic failure in neutrophil-depleted mice. **a**, The 15-passage strategy using the pneumonia model in both immunocompetent and neutrophil-depleted mice. Neutrophil depletion was induced by two doses of cyclophosphamide at 72 and 24 h before infection. At the time of infection, *A. baumannii* ATCC 17978 was oropharyngeally inoculated into mice at 10^8^ c.f.u., and two doses of CIP were administered at 9 and 19 h post infection. All lineages were derived from a single broth-grown culture. At 27 h post infection, the mice were euthanized, lungs were homogenized and bacteria were plated on LB agar plates. After overnight growth, the bacteria were collected and 10^8^ c.f.u. used for the next round of infections/passages. OP, oropharyngeal aspiration; IP, intraperitoneal injection; SC, subcutaneous injection. **b**, Rapid development of antibiotic failure resulting from passage in the absence of neutrophils. After lungs were homogenized, total c.f.u. on LB agar in the absence of antibiotic were determined and plotted as a function of passage number. Shown are three lineages for immunocompetent and neutrophil-depleted animals treated with CIP as described in **a**. Illustrations in **a** were created with BioRender.com. Source data

We serially passaged *A. baumannii* 15 times within a mouse pneumonia model to analyse the dynamics and genetic trajectories of resistance to the FQ antibiotic ciprofloxacin (CIP), with the purpose of determining whether neutrophils play a role in suppressing drug resistance (Fig. 1a). The CIP^S^ (ciprofloxacin susceptible) ATCC reference strain 17978 (AB17978) was passaged by oropharyngeal inoculation in either immunocompetent animals or those depleted of neutrophils by pretreatment with two doses of cyclophosphamide. It should be noted that this treatment also has other effects on the immune system, including the possible reduction of monocyte populations, as well as suppressing T-cell numbers^23,24^. Mice in three parallel lineages were CIP treated at 7 and 19 h post infection (hpi) and bacteria were collected from the lungs of animals euthanized at 27 hpi. The enriched bacterial pools from each independent infection were used as inocula for the next round of infection. The dynamics of bacterial yield was assessed from lung homogenates, and the CIP minimum inhibitory concentration (MIC) of each population was determined, followed by whole-genome sequencing of the heterogenous pool (mean genome coverage depth: 368.4).

### Persistent neutropenia results in treatment failure

A striking result from the passage experiments is that the protocol allowed drug-resistant mutants to overgrow during passage in neutrophil-depleted animals, while the resistant variants in the immunocompetent animal failed to reach high abundance in the population. After each mouse passage in the presence of CIP, the efficiency of bacterial colonization of the lungs was monitored by quantifying total bacterial colony forming units (c.f.u.) in the absence of the drug. Bacterial lung yields increased by 1,000-fold in CIP-treated neutrophil-depleted mice over the course of 15 passages, as compared with little increase in immunocompetent mice (Fig. 1b). After each passage, we observed 10-fold higher colonization in the neutrophil-depleted compared with immunocompetent mice. By passages 5–7, the lung bacterial load in the neutrophil-depleted mice reached the size of the initial inoculum (10^8^ c.f.u.), and animals showed substantial signs of disease, such as lacrimation, piloerection and decreased mobility, despite being CIP treated (Fig. 1b).

**Fig. 2:**
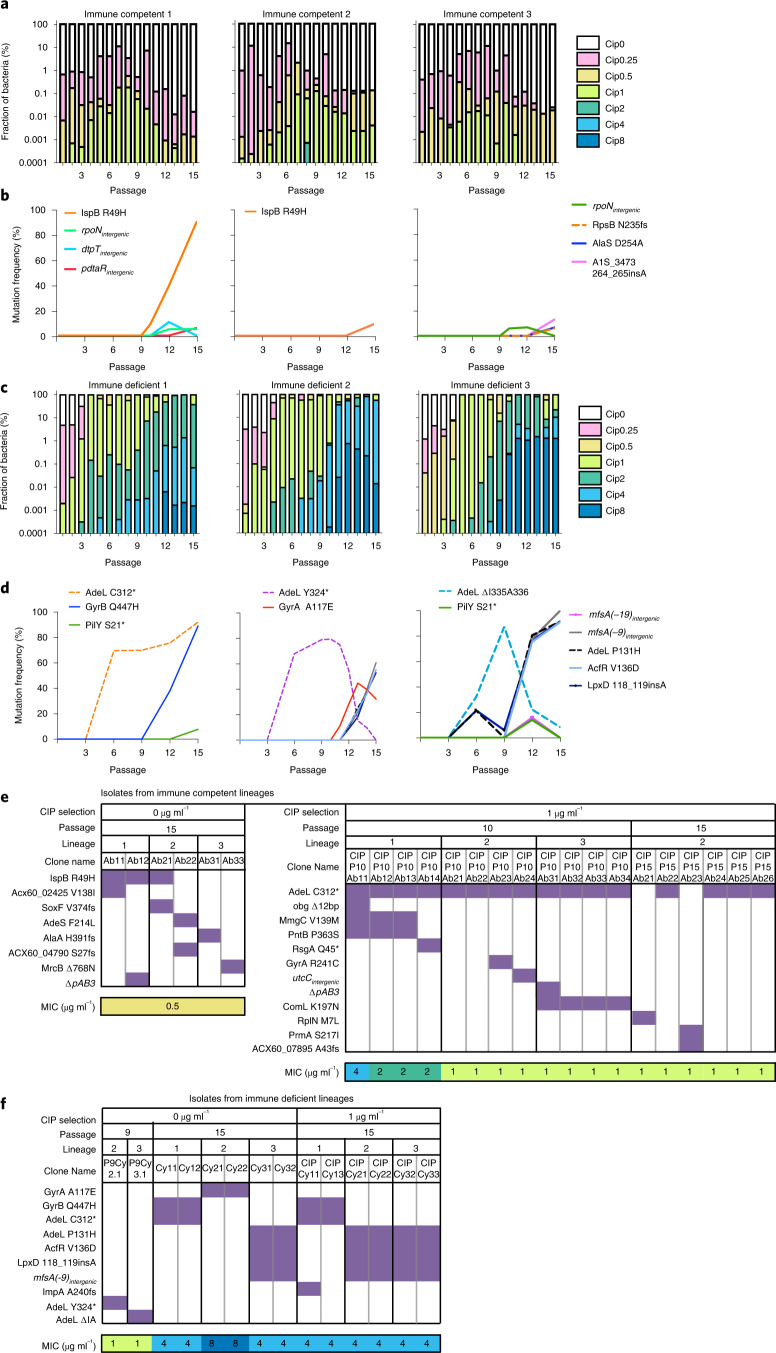
High-level ciprofloxacin resistance evolves in a stepwise fashion during passage in neutrophil-depleted mice. **a**,**c**, After each passage, bacteria from lung homogenates were serially diluted onto LB agar containing noted concentrations of CIP (μg ml^−1^). The fraction of c.f.u. on the series of CIP plates was determined for each pool and displayed in stacked bar plots for immune competent (**a**) and immune depleted (**c**) mouse infection lineages. The limit of detection was 2.7 × 10^−6^. **b**,**d**, The gDNA from each bacterial pool was isolated and subjected to whole-genome sequencing to identify the genomic mutations acquired throughout passaging (after passages 3, 6, 9, 12 and 15). The mutations were detected using a 5% abundance cut-off and filtered against the parent strain AB17978. The relative abundance of each mutation was plotted as a function of passage number from immune competent lineages (**b**) and immune deficient lineages (**d**). **e**,**f**, After 10 or 15 passages (as noted), the bacterial pools from each lineage were incubated on LB in the presence or absence of CIP (1 μg ml^−1^) to select for single colonies. Purified single colonies were subjected to whole-genome sequencing and MIC assays. Each column represents a single colony. Rows display absence or presence of selection criteria, passage, amino acid or SNP (single nucleotide polymorphism) alleles, and MIC values for each single colony isolate. The presence of each mutation in an individual colony is highlighted in purple. MICs are labelled and colour-coded for all colonies, and at least 3 biological replicates were performed. Source data

To determine levels of CIP resistance after each passage, the saved bacteria were quantified on solid medium in the absence or presence of increasing amounts of drug, plotting the fraction of surviving c.f.u. as a function of CIP concentration (Materials and Methods; Fig. 2a,c). Clinically resistant bacteria (CIP ≥ 2 ug ml^−1^) did not arise in the three lineages passaged in immunocompetent mice. Furthermore, the number of bacteria able to survive on increased amounts of CIP relative to the parental strain never rose above 0.1% of the population (survival on solid medium containing 1 ug ml^−1^ CIP). Therefore, in immunocompetent mice, 15 passages were insufficient to allow outgrowth and fixation of resistant variants in the population.

The evolutionary trajectories through neutrophil-depleted mice contrasted strongly with those in immunocompetent mice, as the whole population in all three lineages became CIP resistant over the course of 15 passages in the former. Bacteria resistant to at least 2 μg ml^−1^ CIP were observed as early as the 3rd passage (Fig. 2c). After the 9th passage, approximately 1 × 10^−5^ of the bacteria isolated from the neutrophil-depleted mice showed resistance to 8 μg ml^−1^ CIP, four times higher than the clinically defined resistance breakpoint. By 15 passages, almost all bacteria from the neutrophil-depleted lineages were clinically defined as CIP resistant (at concentration of at least 2 μg ml^−1^), with a large fraction showing resistance to CIP = 8 μg ml^−1^. Therefore, resistance acquisition during disease is amplified in the absence of neutrophils.

### Clinical failure from drug-sensitive bacteria

By the 6th passage in neutrophil-depleted mice, yields of bacteria began to approach those observed in the absence of antibiotic (Fig. 1b), even though the majority of the population remained sensitive to CIP based on the defined clinical breakpoint (Fig. 2c; clinical breakpoint CIP > 2 μg ml^−1^). Yields remained high in successive passages, while the distribution of the population increasingly skewed towards variants with increasing levels of resistance to CIP. Therefore, drug treatment failure at passage 6 under neutrophil-depleted conditions was dominated by bacteria that grew at 1 μg ml^−1^ CIP but showed low viability at the 2 μg ml^−1^ clinical breakpoint. These mutants were replaced by more resistant isolates during later passages (Fig. 2c).

To link specific mutational changes over time to these observed phenotypes, we sequenced the pools of genomic DNA (gDNA) isolated from the original inoculum, the parent AB17978 strain, and the pools collected after the 3rd, 6th, 9th, 12th and 15th passages. Mutation frequencies were then plotted for each passage in the two immune conditions (Fig. 2b,d). On the basis of the predicted sensitivity from the depth of pooled whole-genome sequencing, we expected that only nucleotide changes in greater than 5% of the population could be detected using this approach (Materials and Methods).

The results from the pooled genomic sequencing allowed us to identify changes associated with either the development of drug resistance, or the lack thereof, in these populations. As predicted from Fig. 2a and the sensitivity limits of the analysis, there was no evidence for mutations driving drug resistance at greater than 5% abundance in the three populations evolved in immunocompetent mice. The only alteration that approached fixation was a mutation in a gene encoding a putative solanesyl diphosphate synthase (ACX60_03850; *ispB*), but this allele was unrelated to resistance, as single colony isolates showed no increased survival in the presence of CIP (Fig. 2e). All the other mutations identified by sequencing the pools appeared unrelated to resistance, as none survived incubation on solid medium containing 1 μg ml^−1^ CIP (Fig. 2b,e). Furthermore, the analysis of individual colonies isolated after plating bacterial populations on antibiotic-free medium after the 15th mouse passage identified isolates with CIP MICs that were only slightly elevated relative to that of wild type (WT) (Fig. 2e, left panel; WT MIC = 0.25–0.5).

Variants with altered CIP sensitivity arose in the immunocompetent mice, but they remained at low levels in these populations, as predicted by the previous mathematical modelling study (Fig. 2a)^18^. Mutations causing decreased drug sensitivity were identified by isolating single colonies on solid medium containing 1 μg ml^−1^ CIP, and all 12 of the single colonies selected from the 10th passage had the identical mutation in *adeL*, the regulator of the AdeFGH egress pump^25,26^. The presence of the mutation in multiple lineages is consistent with its existence in the initial bacterial culture before the first inoculation of all three lineages. Interestingly, 8 distinguishable lineage-specific mutations were identified, these mutations being derived from the parental *adeL*(C312*) mutation, consistent with downstream mutations arising during mouse passage (Fig. 2e, right panel). These strains appeared to have been largely lost by passage 15 in the immunocompetent mice (Fig. 2a,e). We conclude that although strains with mutations that activate the AdeFGH egress pump are detected, they are unable to overgrow the WT and are eventually depleted during later passages in immunocompetent mice (Fig. 2b).

Consistent with the dynamic nature of resistance acquisition in neutrophil-depleted mice (Fig. 2b), pool sequencing uncovered mutations that drove the stepwise trajectory to CIP^R^ (ciprofloxacin resistant) in later passages (Fig. 2d). Unlike the immunocompetent lineages, first-step mutations that occurred within *adeL* after 6 passages in neutrophil-depleted mice were easily detected by whole pool sequencing. Each lineage harboured different *adeL* mutations in these early passages, these mutations disrupting the 3’ end of the gene, consistent with previous work arguing that alterations in the C terminus of the AdeL protein result in AdeFGH pump activation^25,26^. Mutations in various other genomic sites arose between passages 9 and 12, with many predicted to contribute to decreased susceptibility to CIP treatment. For instance, the *adeL*(C312*) mutant acquired a second mutation in *gyrB* that overgrew lineage 1. In addition, a mutant harbouring a non-canonical mutation in *gyrA* arose in lineage 2, this mutant being outcompeted by a strain with multiple genetic alterations, while a quadruple mutant appeared to overgrow the single *adeL* mutant in lineage 3.

To identify the various genotypes linked to increased CIP resistance, we isolated single colonies after 9 passages to verify that two transiently predominant variants had the predicted genotypes, and also sequenced isolates from passage 15 after plating in the absence or presence of CIP (1 µg ml^−1^). Isolated colonies in the absence of drug selection from lineages 2 and 3 from passage 9 showed the predicted *adeL* alleles (compare Fig. 2d and f). In addition, after plating the passage 15 pools in the absence of the drug, the two predicted gyrase alleles *gyrA*(A117E) and *gyrB*(Q447H) that are observed infrequently in the clinic were also identified (Fig. 2f). Having *gyrA*(A117E) alone was able to raise the CIP MIC by 16-fold (Fig. 2f). Clones having the *gyrB*(Q447H) allele were only observed to be linked to *adeL*(C312*) at passage 15, resulting in a CIP MIC that was 8-fold higher than that of the parent strain (Fig. 2e). Besides the mutations in target proteins, other single colonies that resulted in a similar MIC increase were found to have a combination of four mutations (Fig. 2f). When comparing the isolated single colonies that had a CIP of 1 μg ml^−1^ to those isolated without antibiotic, the single colony isolates had genomic changes that were predicted by the sequencing of the pools, indicating that the mutants described here became predominant without requiring CIP selection ex vivo.

### Mutations in *adeL* allow for antibiotic persistence

**Fig. 3:**
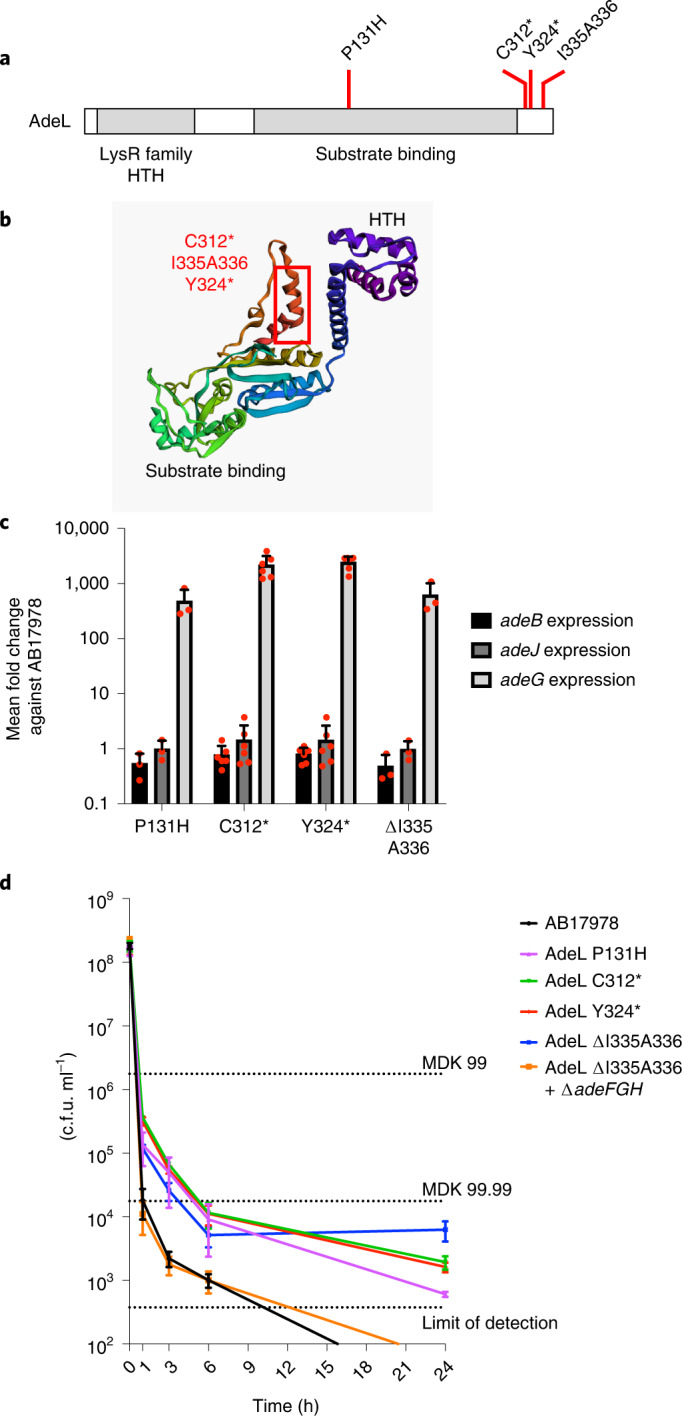
*adeL* mutations drive bacterial persistence through overexpression of the AdeFGH pump. **a**, Sites of non-synonymous changes during in vivo passaging and domain structure of AdeL. **b**, Locations of non-synonymous changes on predicted functional domains of AdeL. Functional domains were predicted using trRosetta^80–82^. Red rectangle represents the domain where three mutations (in red) are located. **c**, Mutations in *adeL* selected during mouse passage result in specific overproduction of an AdeL-regulated gene *adeG*. Data were determined by RT-qPCR analysis of pump-encoding components *adeG* (regulated by AdeL) or *adeB* and *adeJ* (components of other pumps) in noted *adeL* mutant backgrounds. Data shown are individual datapoints (red dots), with bar plot showing mean + s.e.m. At least 3 biological replicates were performed. **d**, Overproduction of AdeL-regulated pump components results in increased persistence in the presence of CIP. AB17978 derivatives described in the legend were exposed to CIP at 20× MIC for noted times and titred for viability by quantitating c.f.u. per mL. Data shown are mean ± s.e.m., and at least 3 biological replicates were performed. Dotted lines represent the minimal duration of killing at 99 (MDK99), 99.99 (MDK99.99) or at the limit of detection for the assay. MDK99, minimum duration of antibiotic exposure at or above the MIC required to kill 99% of the initial bacterial population; MDK99.99, minimum duration of antibiotic exposure at or above the MIC required to kill 99.99% of the initial bacterial population; limit of detection, 375 c.f.u. ml^−1^. Source data

To deconvolve the function of the various mutations observed, we backcrossed mutations into the parent strain and assessed their relative contributions to CIP resistance. Of particular interest were *adeL* nucleotide changes identified in populations after the 6th passage in neutrophil-depleted mouse lineages, as CIP failed to efficiently restrict growth in the lung in these passages (Figs. 1a and 2d). The *adeL* gene (ACX60_06025)^26^ has two predicted domains often associated with LysR-type transcriptional regulators: a helix-turn-helix (HTH) domain and a substrate binding domain responsible for regulation of the AdeFGH pump (Fig. 3a). Of the *adeL* mutations that arose during the in vivo passages, one is located within the predicted substrate binding domain, while the others are at the C-terminal end comprising in-frame deletions or early termination codons (Fig. 3a,b). We constructed strains with each of these mutations and observed 100–1,000× increased *adeG* transcription levels compared with the parental strain, consistent with AdeFGH efflux pump overexpression (Fig. 3c).

**Fig. 4:**
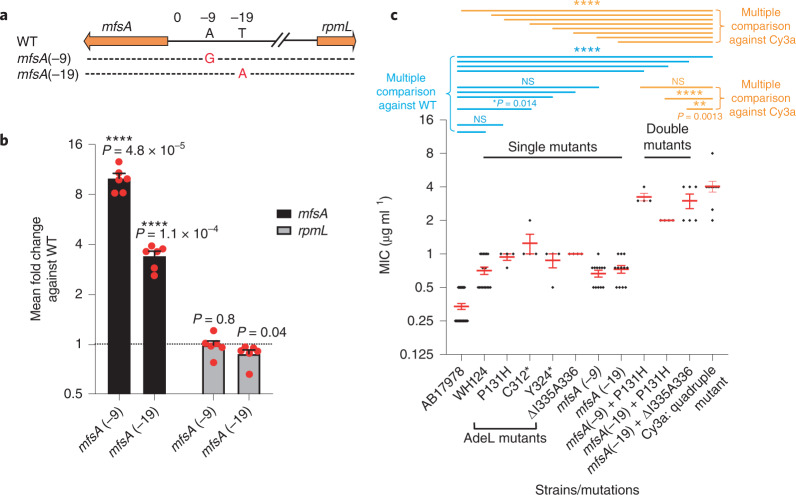
The presence of mutations in *adeL* and *mfsA* is sufficient to explain CIP^R^ in strains derived from mouse passage experiments. **a**, Two mutations located directly upstream of *mfsA* selected during mouse passaging experiments. **b**, Transcription levels of *mfsA* and *rpmL* were determined by RT-qPCR in strains harbouring the two mutations upstream of *mfsA*. ****P < 0.0001 using two-tailed *t*-test against mean fold change of WT. At least 3 biological replicates were performed. Red dots are individual datapoints and bar plots show mean fold change + s.e.m. **c**, Double mutants containing *adeL* and *mfsA* mutations show MICs of CIP that are similar to evolved quadruple mutant strain. Datapoints in black are individual MIC values for each biological replicate, and red bars are mean MICs ± s.e.m. At least 3 biological replicates were performed. WH124: AB17978 with *lpxD* insA *acfR*(V136D) *adeL*(P131H) (*P* = 0.1876 against WT; *P* < 0.0001 against Cy3a); P131H: AB17978 with *adeL*(P131H) (*P* = 0.3382 against WT; *P* < 0.0001 against Cy3a); C312*: AB17978 with *adeL*(C312*), Cysteine to stop codon at residue 312 encoded by *adeL* (*P* = 0.014 against WT; *P* < 0.0001 against Cy3a); Y324*: AB17978 with *adeL*(Y324*), Tyrosine to stop codon at residue 324 encoded by *adeL* (*P* = 0.5137 against WT; *P* < 0.0001 against Cy3a); ∆I335A336: AB17978 with *adeL*(∆I335A336) (*P* = 0.2037 against WT; *P* < 0.0001 against Cy3a); *mfsA*(-9): AB17978 with *mfsA*(-9) (*P* = 0.5744 against WT; *P* < 0.0001 against Cy3a); *mfsA*(-19): AB17978 with *mfsA*(-9) (*P* = 0.2989 against WT; *P* < 0.0001 against Cy3a); *mfsA*(-9)+P131H: AB17978 with *mfsA*(-9) and *adeL*(P131H) (*P* < 0.0001 against WT; *P* = 0.1005 against Cy3a); *mfsA*(-19)+adeL(P131H): AB17978 with *mfsA*(-19) *adeL*(P131H) (*P* < 0.0001 against WT; *P* < 0.0001 against Cy3a); *mfsA*(-19)+ *adeL*(∆I335A336): AB17978 with *mfsA*(-19) *adeL*(∆I335A336) (*P* < 0.0001 against WT; *P* = 0.0013 against Cy3a); Cy3a: *P* < 0.0001 against WT. Statistical significance was tested using one-way ANOVA followed by Dunnett’s multiple comparison. Blue, multiple comparison against WT AB17978 MICs; orange, multiple comparison against MICs of Cy3a quadruple mutant. *P < 0.05, **P < 0.01, ****P < 0.0001; NS, not significant. Source data

While these single *adeL* mutations resulted in an increase in MIC to ~1 μg ml^−1^ CIP, which barely rose to the level of significance (Fig. 4c), this level was below the recognized clinical breakpoint^27^, and such strains would be indicated as susceptible. As these are first-step mutations, we hypothesize that variants harbouring these changes may facilitate the outgrowth of more resistant isolates by promoting tolerance or persistence in the presence of antibiotic. To test this model, we evaluated the survival of *adeL* mutants during exposure to high levels of CIP^28–32^. Exposure to 10 μg ml^−1^ CIP, roughly 20× the MIC of the parental strain, led to the majority of the *adeL* mutants (>99%; MDK99) being rapidly killed (within 1 h), mimicking what was observed for the parental strain (Fig. 3d). However, unlike the parental strain, a subpopulation of the *adeL* mutant was able to persist through 24 h of drug exposure (Fig. 3d). This phenotype was dependent on pump overproduction, as an *adeL* mutant strain deleted for the *adeFGH* operon was indistinguishable from the parental strain in regards to persistence (Fig. 3d). These data argue that *adeL* mutations drive persistence in the presence of CIP via increased expression of *adeFGH*, providing a reservoir within neutrophil-depleted animals for the outgrowth of strains with increased resistance^33^.

### Resistance can be explained by *adeL* combined with *mfsA*

We continued backcross analysis to identify the minimal determinants necessary to confer clinical resistance during passage in the mouse. Many of the single colony isolates from lineage 3 at passage 15 of the immunocompromised mice possessed a combination of mutations in four genomic locations: *adeL*, *acfR* (ACX60_03155), *lpxD* and the intergenic *rpmL*-*mfsA* region, the latter being located between a ribosomal protein gene and a predicted coding region for a putative MFS transporter, which we have named *mfsA* (ACX60_15150, annotated as *nreB* in GenBank; Fig. 2d,e). To determine the role of each of these mutations in drug susceptibility, we constructed separate strains carrying single mutations. All single mutants showed various MICs well below 2 μg ml^−1^ CIP, with each of the *adeL* mutations as well as *mfsA*(−9) showing increased MICs above the parental strain (Supplementary Table 1 and Fig. 4c). The *mfsA*(−9) mutation is located 9 bp upstream of a predicted MFS efflux pump, within 2 bases of a previously reported mutation selected during evolution of CIP resistance in *A. baumannii* in planktonic conditions^34^. Consistent with MFS transporter overproduction, we found that the *mfsA*(−9) mutation increased the *mfsA* mRNA levels by ~9-fold relative to the parental strain, on the basis of q-rtPCR analysis (Fig. 4b). Significantly, when the *mfsA*(−9) mutation was combined with the *adeL*P131H mutation, the CIP MIC increased to ~4 ug ml^−1^, approaching that of the quadruple mutant strain isolated from mouse passage 15 (Fig. 4c and Supplementary Table 1). A single nucleotide change, located near the *mfsA*(-9) mutation, that arose transiently in the immunodepleted lineage 3 (Fig. 2d; *mfsA*(-19)) behaved nearly identically (Fig. 4b,c). Therefore, combining two mutations that resulted in upregulation of both the AdeFGH pump and a putative MFS transporter was sufficient to provide CIP resistance above the clinical breakpoint, approaching that seen for the quadruple mutant.

### Mutations in *lpxD* is linked to fluoroquinolone resistance

**Extended Data Fig. 1:**
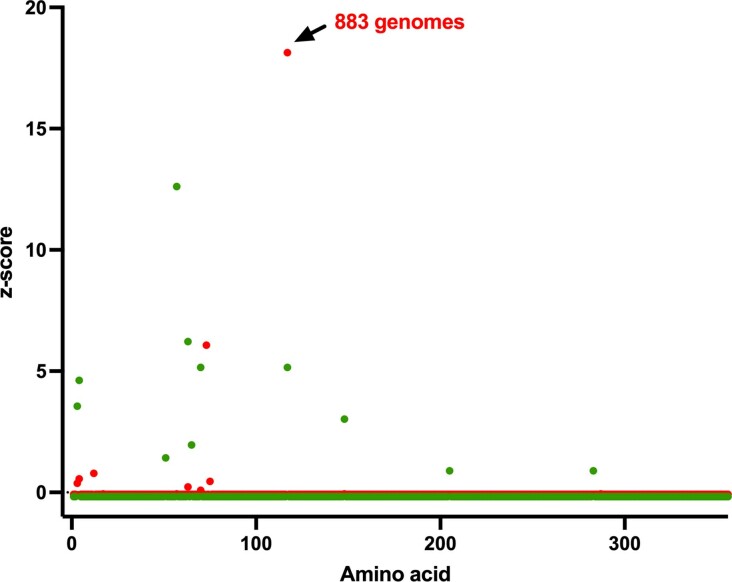
The LpxD E117K allele is tightly linked to clinical fluoroquinolone resistance. Assembled genomes of 2045 clinical isolates were obtained from PATRIC database^80^. Predicted LpxD sequences from all genomes were aligned and compared to ATCC 17978 and the total number of variants per defined amino acid was calculated for each group of genomes. The total number of amino acid changes was normalized and presented as a z-score for each of the three groups, plotted against the residue numbers.

The mutation *lpxD*(T118_A119insA) (locus tag: ACX60_07955; GenBank: AKQ26661.1) was consistently found in CIP^R^ isolates from mice. This alteration within an acyltransferase involved in lipo-oligosaccharide biosynthesis^35–37^ showed no effect on MIC (Supplementary Table 1). To test for clinical linkage of *lpxD* mutations to drug resistance, we downloaded the genomes of all 8,666 *A. baumannii* genome clinical isolates from the PATRIC database^38^. Focusing on non-repetitive sequences that had antibiotic resistance profiles, 1,830 were resistant to ciprofloxacin and 215 were susceptible. Of the CIP^R^ isolates, 883 genomes had an E- > K change at residue number 117, with a *z*-score ≥ 18 (Extended Data Fig. 1). In comparison, only 9 of 215 CIP^S^ isolates had the same mutation. By Fisher’s exact *t*-test, *P* < 0.0001, supporting the significance of these observations.

**Fig. 5:**
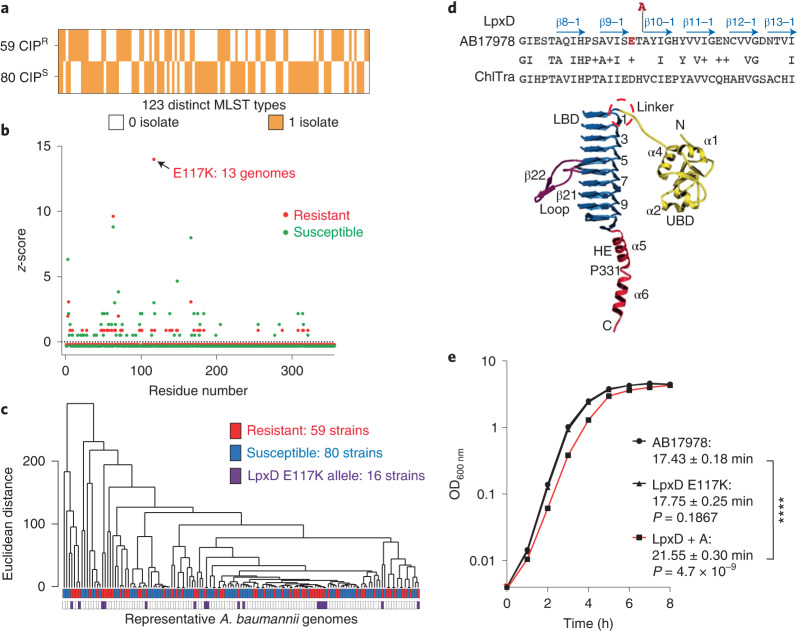
The LpxD E117K allele is tightly linked to clinical fluoroquinolone resistance. **a**, Assembled genomes of 139 clinical isolates with ciprofloxacin resistance profiles from the PATRIC database^83^. Displayed is a heatmap showing the presence (orange, 1 isolate) or absence (white, 0 isolate) of clinical isolates for each ST per resistance group. **b**, LpxD sequences from resistant (red dots) and susceptible isolates (green dots) were aligned and compared to ATCC 17978, and the total number of variants per residue was calculated for each group of genomes. The total number of amino acid changes was normalized and presented as a *z*-score for each group, plotted against the LpxD residue number. **c**, Hierarchical clustering of the 7-gene MLST profile from 139 genes showing Euclidean distance as height and nodes colour-coded according to resistance profile (red, CIP^R^; blue, CIP^S^) and the presence of the *lpxD*(E117K) allele (purple). **d**, Structure of *Chlamydia trachomatis* LpxD protein^38^ (Copyright (2007), National Academy of Sciences, USA) showing the presumed site of the *A. baumannii* E117 based on sequence similarity. Red highlighted E, E117; red highlighted A, insertion of A between residue 118 and 119; blue arrows, beta sheets. **e**, Broth growth of AB17978 strains differing by single changes in *lpxD*. Shown are strains harbouring either the WT, *lpxD*(T118A119_insA) or *lpxD*(E117K) alleles. Four independent biological replicates were performed. Statistical significance was determined for doubling time using one-way ANOVA followed by Dunnett’s multiple comparison. ****P < 0.0001 for *lpxD* + A strain against WT. Data shown are mean ± s.e.m. Source data

**Extended Data Fig. 2:**
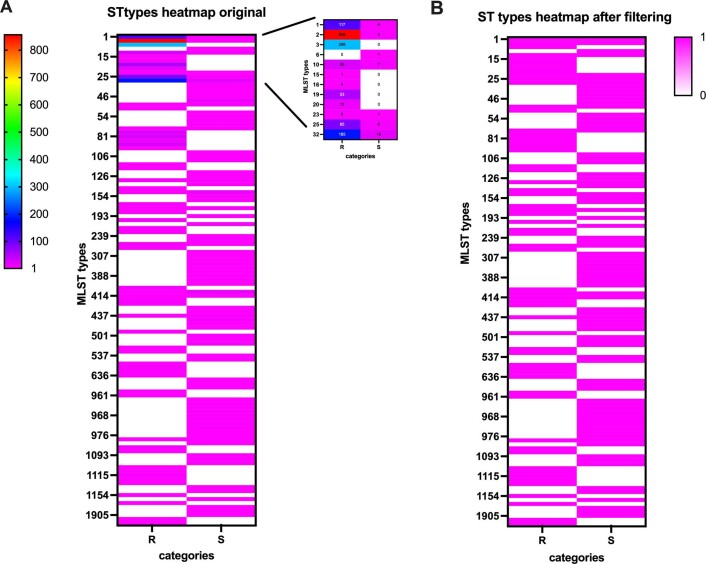
Distribution of MLST type among select clinical isolates. A) Heatmap showing MLST enrichment among 1830 genomes from ciprofloxacin resistant isolates and 269 genomes from susceptible isolates. In order to reduce the populational clonal effect, analysis was limited to only one representative genome per ST group. Heatmap in B) shows the distribution of MLST types after filtering.

**Extended Data Fig. 3:**
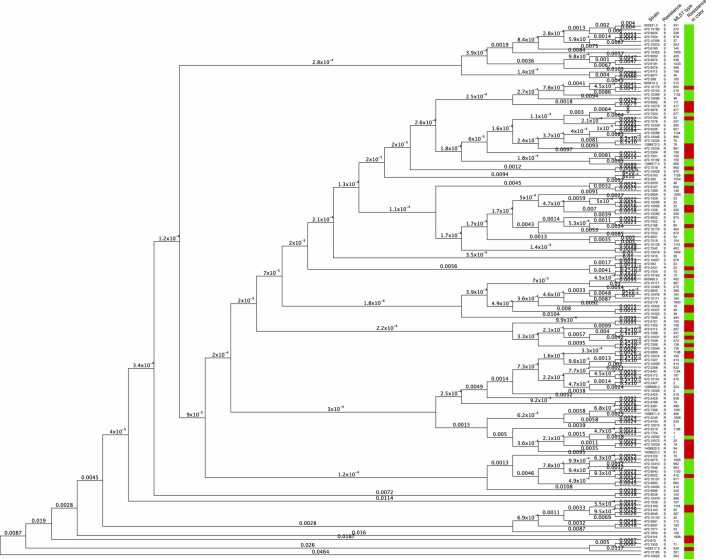
Phylogenetic tree of genomes from 139 clinical isolates. The genome contigs were analyzed to build the phylogenetic tree using mashtree with the minimum abundance finder.

To determine whether the overrepresentation of the *lpxD*(E117K) allele could be due to outgrowth of a single clone, we performed multilocus sequence typing (MLST) using the Pasteur *A. baumannii* scheme, generating 7-gene allele profiles^39^. Of the 123 distinct MLST groups, 16 had both CIP^R^ and CIP^S^ isolates (Supplementary Dataset 1 and Extended Data Fig. 2), although over half of the CIP^R^ isolates belonged to two MLST types (ST2 and ST3). To avoid skewing the results due to this overrepresentation, we limited the number of isolates per ST group to 1, reducing strains analysed to 139 across 123 distinct MLST groups (Fig. 5a), including 59 CIP^R^ isolates and 80 CIP^S^. Even after limiting the clonal effects in this fashion, we still observed overrepresentation, with the E117K allele found in 13 of 59 CIP^R^ isolates (Fig. 5b), while only 3 of 80 CIP^S^ isolates had the same alterations (Fig. 5b; *P* = 0.0011, Fisher’s exact *t*-test). Both a whole-genome phylogenetic tree (Extended Data Fig. 3) and clustering analysis of the 7-gene allelic profiles from these 139 clinical isolates (Fig. 5c) identified CIP^R^ isolates that shared parents with CIP^S^ ones, further arguing against clonal effects for the enrichment.

Interestingly, the alteration at residue E117 (*z*-score, 8.98; *P* < 0.00001) directly precedes the T118 allele identified in our mouse experiments, with both variants predicted to be in a turn between two beta sheets (Fig. 5d; Copyright (2007), National Academy of Sciences, USA)^38^. Although the significance of this linkage is unclear, the fact that the *lpxD*(T118_A119insA) has a fitness defect in broth (Fig. e) raises the possibility that mutations in this turn could contribute to a persistence phenomenon in tissues, leading to evolution of drug resistance.

### Evolutionary replay reconstructs drug resistance pathway

**Fig. 6:**
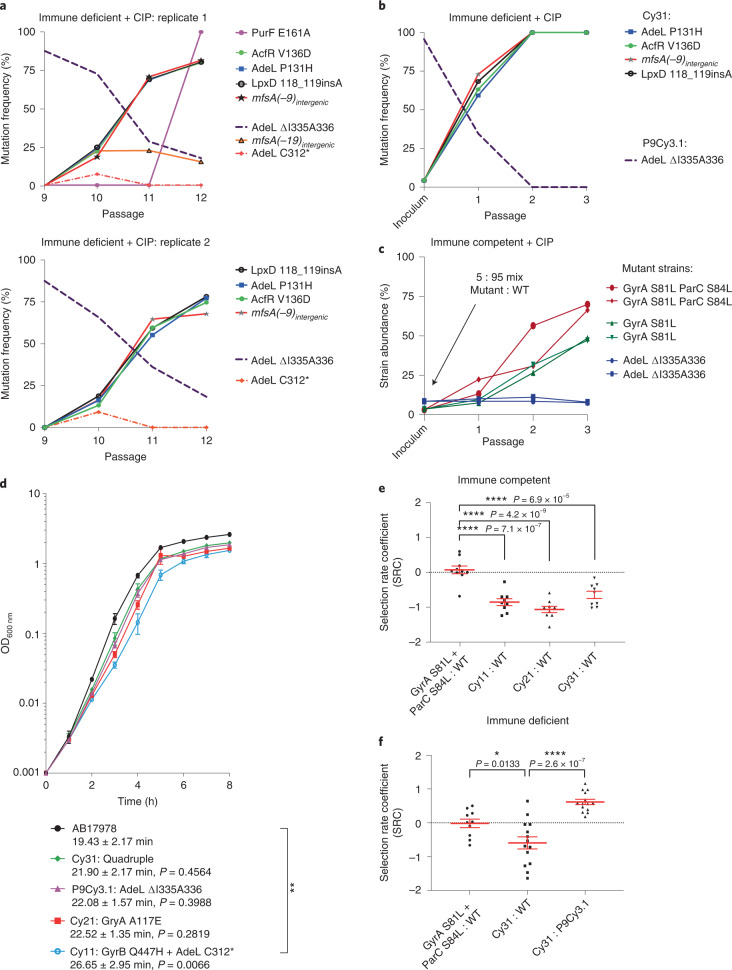
Fitness advantage in the presence of drug drives evolutionary trajectory. **a**, An *adeL* mutant is outcompeted by a quadruple mutant (Cy31) having drug-resistant *adeLmfsA* alleles during drug treatment. Passage 9 pool was oropharyngeally inoculated into neutrophil-depleted mice in separate lineages and passaged 3 times in the presence of CIP. **a**,**b**, Plots showing abundance of mutations identified in pools as a function of the passage number. Strains were mixed at a ratio of 95:5 (single:quadruple mutant), and oropharyngeally inoculated into neutrophil-depleted mice in the presence of CIP. Deep sequencing was performed to determine the ratio of strains, with abundance of each mutation displayed as a function of passage. **c**, Presence of neutrophils prevents enrichment of *adeL* mutant during CIP challenge. Competition of *adeL*(ΔI335I336), *gyrA*(S81L) or *gyrA*(S81L) *parC*(S84L) against WT was performed. Strains were mixed at a ratio of 95:5 (WT:mutant), oropharyngeally inoculated into immunocompetent mice, then passaged in separate lineages in the presence of CIP. Abundance of each mutant was determined by measuring the colony forming efficiency (CFE) on CIP at a concentration below the mutant MIC but above that of WT (*adeL*(ΔI335I336), MIC = 0.75 µg ml^−1^; *gyrA*(S81L), MIC = 0.75 µg ml^−1^; or *gyrA*(S81L) and *parC*(S84L), MIC = 2 µg ml^−1^). CFE = colonies on antibiotic-containing medium/colonies in the absence of antibiotic, displayed as strain abundance (%). **d**, Browth culture growth rates of isolates from mouse passage. Data are mean ± s.e.m. from 3 independent biological replicates. Significance determined by one-way ANOVA followed by Dunnett’s multiple comparison. **P < 0.01 for Cy11 against WT. C312*, stop codon at residue 312. **e**,**f**, Neutrophil-replete (**e**) and neutrophil-depleted (**f**) (cyclophosphamide-treated) BALB/C mice challenged oropharyngeally with noted strains for 24 h to determine the selection rates. At least 9 independent biological replicates were performed. Data shown are individual datapoints (black), with red bars showing mean ± s.e.m. Statistical significance was determined by one-way ANOVA followed by Dunnett’s (**e**) or Tukey’s (**f**) multiple comparison. *P < 0.05, ****P < 0.0001. Source data

The observation that resistant organisms outcompeted a persistent strain (Fig. 2d) could be due to a coincidence that occurred during serial passage or due to antibiotic-driven selection for strains with increased MIC over persistent strains. We performed 3 evolutionary replay experiments^40^ with neutrophil-depleted lineage 3 variants to distinguish between these possibilities. In the first (Fig. 6a), bacteria were collected after passage 9 and cycled 3 times in duplicate in neutrophil-depleted animals to test whether the *adeL*(ΔI335A336) allele, a 6-nucleotide in-frame deletion identified in cyclophosphamide-treated animals, would again be outcompeted by low abundance drug-resistant mutants (Fig. 2d). The second approach was to passage 3 times a mix of a colony-purified drug-persistent *adeL*(ΔI335A336) single mutant (P9Cy3.1; Supplementary Table 1) with a colony-purified drug-resistant quadruple mutant (Cy31; Supplementary Table 1) in the approximate ratio present at passage 9 (95:5; Fig. 6b). The third was to test the model that neutrophils restrict the outgrowth of strains having activating mutations in *adeL* (Fig. 6c).

**Extended Data Fig. 4:**
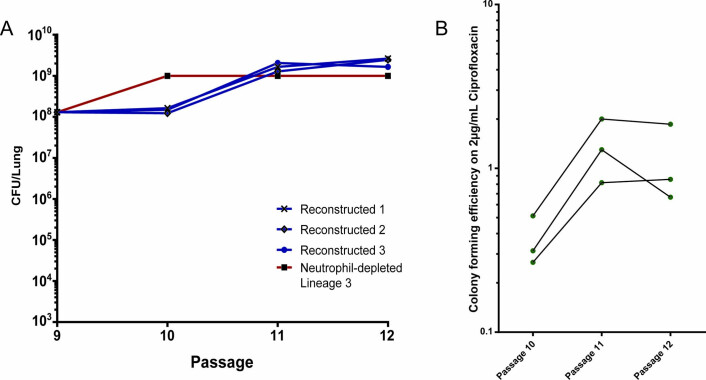
Bacterial load and resistance levels to CIP after re-passaging. A) The bacterial load harvested from the lungs after each passage is plotted against the passage number for each Lineage. B) CIP resistance level of each pool (after each passage) was assayed via quantification of CFU on LB agar supplemented with CIP.

When the *adeL* mutant was analysed in competition within the pool (Fig. 6a) or when mixed 95:5 with colony-purified quadruple mutant (Fig. 6b), we observed an increase in bacterial burden and the fraction of bacteria showing CIP MIC > 2 after passaging the pools in neutrophil-depleted CIP-treated mice (Extended Data Fig. 4). Whole-genome sequencing of the bacterial populations collected after each passage revealed a near-identical evolutionary trajectory as seen from the original experiment, with the single mutant associated with drug persistence eventually outcompeted by drug-resistant mutants (Fig. 6a–c). Furthermore, in one of our replicate lineages (Fig. 6a), another mutation located in the intergenic region between *rpmL* and *mfsA* (*mfsA*(-19)) appeared transiently in immunodepleted lineage 3 (Fig. 2d), further arguing for the contribution of this regulatory region in resistance evolution. Therefore, these two reconstructions (Fig. 6a,b) demonstrate that the evolutionary trajectory is reproducible, and can be regenerated by phenotypically similar mutations that arise spontaneously.

In the third experiment, single *adeL* mutants were unable to overgrow the WT in the presence of CIP in immunocompetent animals, in contrast to what was observed in passage 9 of the neutrophil-depleted animals. When a 5:95 (*adeL*:WT) mixture was passaged in immunocompetent mice, the mutant was unable to increase its population share (Fig. 6c), mimicking the failure of *adeL* activating mutations to overgrow the pool in any of the immunocompetent lineages (Fig. 2a). In contrast, CIP^R^ mutants having canonical resistance alleles in *gyrA* or *gyrAparC* were able to compete efficiently with WT in the presence of neutrophils, with the *gyrA*(S81L) *parC*(S84L) double mutant growing from 5% to 70% of the population within 3 passages (Fig. 6c). This argues that depleting the immune response allows the outgrowth of persister mutants, facilitating complex pathways to drug resistance that are blocked in immunocompetent hosts.

### Ciprofloxacin treatment selects for enhanced fitness

Antibiotic resistance is often associated with a fitness cost^41^ that may be compensated over time by continued selection of secondary genotypes that overcome these costs. The inability of the *adeL* mutant to overgrow WT during infection in the presence of CIP in immunocompetent animals may reflect this point. Mutants with high resistance, however, could maintain their selective advantage over non- or low-resistance mutants even in the absence of drug treatment. To test this model, the relative fitness of the drug-resistant mutants derived from neutrophil-depleted lineages was compared to that of the parent strain AB17978. In the absence of CIP, variants isolated after passage in neutrophil-depleted mice showed varying degrees of subtle growth impairment in broth relative to the parental strain (Fig. 6d; Cy11, *P* < 0.01). During animal infections in the absence of antibiotic, these growth defects were amplified. When immunocompetent mice were challenged with the high fitness *gyrAparC* mutant in competition with WT, the two strains showed similar levels of colonization (Fig. 6e). In contrast, each of the drug-resistant mutants derived from neutrophil-depleted mice competed poorly in immunocompetent animals (Fig. 6e; *P* < 0.0001).

Although the lowered fitness of mutants in immunocompetent animals further supports the model that outgrowth of unique resistance alleles occurs in the absence of neutrophils, it did not directly test the model that selection in neutropenic mice increased fitness relative to first-step CIP^R^ variants. In the absence of CIP treatment, the parent strain showed a subtle advantage over the lineage 3 quadruple mutant isolate Cy31 (Supplementary Table 1) under neutrophil-depleted conditions, although the results were clearly not as robust as in the presence of neutrophils due to increased scatter (Fig. 6f; Cy31 against WT). More striking is the fact that the Cy31 strain had a clear fitness advantage over its predecessor, the lineage 3 *adeL* mutant, when competed in the absence of antibiotics (Fig. 6f; Cy31 against P9Cy3.1). Therefore, in competition with persister mutants, ciprofloxacin simultaneously selected for an isolate with increased CIP resistance and enhanced fitness relative to mutant competitors. This predicts that drug-resistant mutants can evolve to heightened fitness to replace persister mutants. As fitness increases, resistant mutants should be able to compete with sensitive strains even after cessation of antibiotic treatment, potentially escalating treatment failure and providing fertile ground for the emergence of resistant strains with high fitness.

## Discussion

Comparative analyses of bacterial pathogen databases indicate that clinical antibiotic resistance is associated with mutations located outside well-characterized drug targets^20–22^. Analysis of clinical resistance is often retrospective, with a few exceptional studies that have allowed identification of a timeline of bacterial resistance in human populations^28–32,42–44^. Our study bridges an important gap in understanding how resistance evolves, by following experimental evolution of fluoroquinolone resistance in a pneumonia disease model. Strikingly, in the absence of one arm of immune pressure, *A. baumannii* resistance arose after few passages in murine lungs during CIP treatment (Fig. 1b), resulting in treatment failure. Conversely, the overall frequency remained low in immunocompetent mice, and this did not lead to treatment failure (Fig. 1b).

The absence of neutrophils resulted in the identification of rare alleles associated with drug resistance. In contrast to the canonical target site *gyrA*(S81L)*parC*(S84L) mutant that shows high fitness under conditions tested, the *gyrA*(A117E) and *gyrB*(Q447H) alleles rarely observed in clinical strains predominated in two of the lineages from neutrophil-depleted animals (Fig. 2e). These unusual isolates showed fitness defects during disease and/or in culture (Fig. 6), making them unlikely to arise in the presence of intact immune functions, consistent with evolutionary trajectories being determined by the immunological state of the host. Therefore, we propose that immunocompromised hosts are incubators for the generation of unique drug-resistant variants, with uncertain outcomes after pathogen exposure to the community at large. Although the variants we described have reduced fitness relative to WT, continued passage demonstrated that stepwise increase in drug resistance was associated with stepwise increase in fitness (Fig. 6). Similar fitness effects have been obtained during continued passage in culture^34^. The recent demonstration of enhanced viral evolution of SARS-CoV-2 (the virus that causes COVID-19) in an immunocompromised patient undergoing therapeutic antibody treatment is a graphic example of the potential interplay between immunity, antimicrobials and evolution in the clinic, with potential large-scale community effects^45^. Furthermore, persistence mutants have been demonstrated to arise during continued antibiotic therapy in an immunocompromised patient treated for vancomycin-resistant enterococcus^46^, tying the results presented here with documented clinical outcomes.

Antibiotics primarily act by targeting essential cellular functions or pathways^47,48^. In response to transient antibiotic exposure, a proportion of bacteria typically persist or tolerate this treatment, with mutations arising that can increase the fraction of the surviving population^49–51^. The mechanisms for allowing persistence/tolerance probably differ between bacterial species and vary among antibiotics^52^, but it is generally agreed that persistence/tolerance is caused by cell dormancy^53–55^ or transient expression of efflux pumps and stress response pathways^28,56^. Persistence/tolerance mechanisms may play an important role in the relapse of bacterial infections^57–59^. In our study, we found that persistence in *A. baumannii* can be promoted by upregulation of a drug efflux pump, AdeFGH^26^. It is known that clinical isolates often overproduce efflux pumps that remove multiple antibiotics from the bacterial cytoplasm^26,60^, and that antibiotic persistence is associated with such pump upregulation^61,62^. Of note, mutations upregulating this pump are rarely isolated in the laboratory through simple antibiotic single-step selections on solid agar, indicating that there may be a special environment in the lung that allows outgrowth of these mutants. It should be noted that all lineages in both immunocompetent and the immune-depleted mice were derived from a single bacterial culture. This had the disadvantage of having mutations present in the initial culture to be selected. It also had the unexpected advantage of demonstrating that a mutant restricted in immunocompetent mice was able to proliferate in the absence of neutrophils.

There are three important repercussions from the analysis of the AdeL efflux overproducer mutations. First, overproduction results in drug treatment failure during pneumonic disease in the mouse, even though these bacterial strains are CIP-sensitive based on the international standard for clinical breakpoints^27^. Therefore, clinical drug treatment failure in the absence of identified antibiotic resistance^9^ could be explained by the outgrowth of drug-persistent mutants, particularly in the immunocompromised host. Second, the *adeL* mutations provide a molecular basis for *A. baumannii* to develop second-step variants that lead to CIP resistance above the clinical breakpoint. Of interest, inhibitors of efflux pumps have been suggested to re-sensitize bacteria to multiple antibiotics^63^, but such a regimen might also delay progression to resistance. Finally, isolates from drug treatment failure occurring in immunocompromised patients could generate a pool of precursor mutants giving rise to resistant isolates that eventually infect a broad range of patients. Several studies have similarly argued that efflux pump mutations provide a critical pool for the multistep evolution of antibiotic resistance^33,64,65^

**Extended Data Fig. 5:**
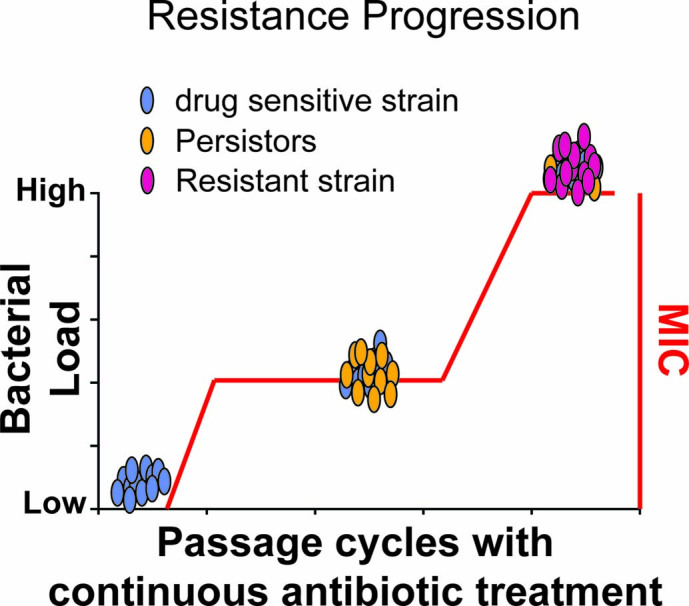
Model of bacterial resistance progression. The antibiotic susceptible inoculum (blue) colonizes poorly in presence of antibiotic. During continued passage with CIP, in the absence of neutrophils, the persister mutants (yellow) arise as first step mutations showing intermediate MICs. With continued passage under the same antibiotic treatment regime, second step mutations arise that have increased fitness in the presence of antibiotic (red) and dominate the population.

We propose a model of bacterial resistance progression in immunodepleted hosts (Extended Data Fig. 5). When infecting a host, the antibiotic-susceptible bacteria colonize at a relatively low rate. With active transmission and continuous CIP treatment, there is outgrowth of bacterial persisters that have MICs below the clinical breakpoint, increasing the bacterial load at the infection site. On continued treatment, or transfer to a similar host undergoing CIP treatment, fully resistant mutants can then outgrow the population. The presence of intact immune function interferes with outgrowth of these mutants, but the identification of persister mutants in the presence of immune function indicates that such alleles could act as enablers of drug resistance in all hosts, particularly under conditions of temporary breaching or depression of innate immunity.

Genomic analyses can identify allelic variants linked to drug resistance, but verifying the functional importance of these alleles is hindered by founder effects that are often difficult to discount. The *lpxD*(E117K) mutation is one such allele associated with resistant *gyrA* S81L-containing genomes. The fact that the adjacent *lpxD*(T118 insA) allele was isolated during multiple passages in immune-depleted mice argues for a role of these altered residues in supporting the outgrowth of drug-resistant organisms. The *lpxD*(T118 insA) mutation probably alters envelope function. This raises the possibility that the mutation causes subtle changes in permeability that slow drug access to target, or else the slowed growth of strains harbouring this allele increases drug tolerance during growth in tissues^52^. Our inability to demonstrate increased tolerance to CIP and the fact that the clinical *lpxD*(E117K) did not show a fitness defect when grown in LB broth (Fig. 5e) argues that the critical phenotypes may be observed exclusively during growth in tissues, making them difficult to evaluate.

In summary, we hypothesize that resistance progression in clinical isolates follows a similar trend witnessed in our experiments, albeit with greater complexity in the human. Drug-sensitive bacteria may colonize both healthy and immune system-compromised patients, but resistance evolution occurs more rapidly within hosts having impaired neutrophil function. The canonical resistance mutations that arise within healthy individuals may compete efficiently with host-evolved mutations in patients having impaired neutrophil function. Hence, the transmission from individuals having intact immune function may pose risks to immunocompromised patients. Adding to the risks to vulnerable patients, we found that *A. baumannii* persistence mutants with MIC levels below the clinical breakpoint were associated with treatment failure, emphasizing the difficulty in treating these patients (Fig. 1b)^9,66–69^. While heterogeneity and variability between infected patients is inevitable, this work provides insight into some genetic factors that could predispose individuals to treatment failures in specific clinical contexts. Future work should be focused on how this evolutionary trend towards drug resistance can be controlled, such as tackling the molecular basis for selection of persister or other mutations that lead to treatment failure.

## Methods

### Use of animals and ethics statement

All animals in the study are 6–8-week-old female BALB/C mice. A sample size of 3 was selected for animal experiments to minimize the use of animals. Neutrophil depletion procedure and bacterial infection were performed on randomly selected mice. Use of immunocompromised animals cannot be blinded as they are readily identified by their increased lethargy and disease susceptibility relative to untreated animals. All mice were housed in ventilated caging systems (10–15 air changes per hour) at temperatures of 68–79 °F (~20–26 °C) and 30–70% humidity, with 12 h light/12 h dark cycle. All animal procedures were approved by the Institutional Animal Care and Use Committee (IACUC) of Tufts University. The animal care and use programme at Tufts University/Tufts Medical Center – Boston Campus has been continuously accredited by AAALAC since 18 April 1966. The facility maintains a current USDA research license (14-R-0082) and holds the Public Health Service Policy Assurance number D16-00459 (A3775-01). The animal care and use programme maintains compliance with these regulatory bodies.

### Bacterial strains

Bacterial strains used in this study are listed in Supplementary Table 1. *A. baumannii* strains are derivatives of ATCC 17978. Bacteria were grown in Lysogeny Broth (LB, BD244620) (10 g l^−1^ tryptone, 5 g l^−1^ yeast extract, 10 g l^−1^ NaCl) or on LB agar plates (LB supplemented with 15 g l^−1^ agarose, BD214010). Broth cultures were grown at 37 °C in flasks with orbital shaking at 200 r.p.m. or in tubes with rotation on a roller drum at 56 r.p.m. Growth was monitored by measuring absorbance spectrophotometrically at 600 nm (A_600nm_). Plates were incubated at 37 °C. Antibiotics were used at the following concentrations: Gentamicin (Sigma, G3632), 10 μg ml^−1^ (*A. baumannii*) and 50 μg ml^−1^ (*Escherichia coli*); Carbenicillin (Sigma, C1389), 100 μg ml^−1^.

### Murine experimental evolution

The in vivo passaging experiments were performed identically in either immunocompetent or neutrophil-depleted 6–8-week-old female BALB/C mice (Fig. 1a). Neutrophil depletion was induced via cyclophosphamide pretreatment^70^. Cyclophosphamide monohydrate at 150 mg kg^−1^ and 100 mg kg^−1^ (Sigma-Aldrich: C7397) were administered 4 d and 1 d before infection, respectively. At the time of infection, mice were transiently anaesthetized by inhalation of isoflurane and lung infections were established using ~10^8^ colony forming units (c.f.u.) of *A. baumannii* from mid-log phase cultures (A_600nm_ ~ 0.5) via oropharyngeal aspiration^71^. For passage 1, all lineages were derived from a single broth-grown culture. At 7 and 19 hpi, 100 mg kg^−1^ of ciprofloxacin (Sigma-Aldrich, PHR1044) was administered via subcutaneous injection. At 27 hpi, mice were euthanized, and lungs were removed aseptically and homogenized in 1 ml of ice-cold 1× phosphate buffered saline (PBS; Thermo Fisher, 10010049). Afterwards, the homogenate was plated on large (150 ×15 mm) LB agar plates and incubated for ~17 h at 37 °C. The total number of bacteria was quantified using c.f.u. The enriched bacteria were scraped off the LB plates and resuspended in 1× PBS. The resuspension was used for DNA isolation and as inoculum for the next passage/round of infection (stored at −80 °C in 20% glycerol before use). For each round of mouse infection/passage, frozen bacterial stocks were revived by diluting them in LB broth and growing them to exponential phase. Afterwards, ~10^8^ c.f.u. of the pool was used to establish infection in each mouse. Three separate lineages of murine infections were maintained in parallel in each condition (immunocompetent or neutrophil depleted), and within each lineage, each starting inoculum was passaged 15 times. Validation experiments involving the reconstruction of identified mutants and re-passaging of these strains in mice were similarly performed.

### Murine competition assays

Competition experiments were performed following previously published procedures^71^. Designated strains were grown to exponential phase (A_600nm_ ~ 0.5–1.0) and mixed at a 1:1 ratio for the inoculum. Afterwards, ~10^8^ c.f.u. of the bacterial mixture was used to challenge WT or cyclophosphamide-treated BALB/C mice via the oropharyngeal route as described for the mouse passage experiments. At 24 h post inoculation, lungs were removed aseptically, homogenized and extracts were spread on LB agar plates. To quantify the input and output ratio of each designated strain, both the inoculum and the lung homogenate were serially diluted in 1× PBS and plated on LB agar as well as LB agar supplemented with 2 μg ml^−1^ ciprofloxacin to differentiate between the WT and CIP-resistant test strains. Selection rate coefficients (SRC) were determined as SRC = ln(Strain 1 output/input ratio) – ln(Strain 2 output/input ratio)^72^. GraphPad Prism v9 was used for statistical analysis and significance was determined by one-way analysis of variance (ANOVA) followed by Dunnett’s or Tukey’s multiple comparison. *P < 0.05, ****P < 0.0001.

### Evolutionary replay experiments

For the evolutionary replay experiments (Fig. 6a), the saved Cy3 passage 9 pool was used as inoculum. For the evolution experiments involving isolates (Fig. 6b,c), designated strains were grown to exponential phase and mixed at a 5:95 ratio in the inoculum. In both cases, the inoculum was administered via the oropharyngeal route and passaged three times in the presence of CIP treatment in a near-identical manner as the initial passaging experiments. Each pool/mixture was performed in duplicate. The bacterial pools after each passage were saved, and variant frequencies at each passage were determined as described above. The abundance of each mutant in the isolate mixtures was determined by measuring the colony forming efficiency (CFE) at an antibiotic concentration that was below the mutant MIC but above that of WT AB17978. The CFE versus passage number was displayed and expressed as strain abundance (%). CFE = number of colonies on antibiotic-containing medium/number of colonies in the absence of antibiotic.

### Determining resistance as a function of drug concentration

For each passage, to evaluate the fraction of bacteria that are viable on culturing in various concentrations of ciprofloxacin, the glycerol stock of each pool was revived in fresh LB broth and grown to exponential phase. A total of ~10^7^ c.f.u. was used for serial dilutions in 1× PBS and 10 μl from each diluted culture was spotted on LB agar plates containing the following concentrations of ciprofloxacin: 0, 0.25, 0.5, 1, 2, 4 and 8 μg ml^−1^. After an overnight incubation at 37 °C, colonies were counted, and c.f.u. per ml was calculated. A detection limit of 100 c.f.u. ml^−1^ was used. The fraction of bacteria resistant to a certain concentration (C_0_; C_0_ > 0) of ciprofloxacin was calculated as (c.f.u. ml^−1^ at C_0_ − c.f.u. ml^−1^ at all concentrations above C_0_) / Total c.f.u. A stacked bar plot of the data was generated using GraphPad Prism (Fig. 2).

### Whole-genome sequencing

Genomic DNA was extracted from bacteria using the DNeasy blood and tissue kit (Qiagen, 69506). Library preparation was performed using Illumina Nextera^73^. For each sample, 7 ng of gDNA was fragmented using Illumina Tagment DNA TDE1 enzyme (Illumina, 20034198) for 5 min at 55 °C. P5 and P7 indices (Illumina FC-131-1002) were then ligated to the fragments through 8 cycles of PCR (15 s at 98 °C, 30 s at 60 °C, then 1 min 30 s at 72 °C) using EBNext High-Fidelity 2X PCR Master Mix (NEB, M0541S). The universal sequencing primers (P1: AATGATACGGCGACCACCGA; P2: CAAGCAGAAGACGGCATACGA) were ligated using 4 cycles of PCR (20 s at 98 °C, 20 s at 60 °C, then 30 s at 72 °C) using EBNext High-Fidelity 2X PCR Master Mix (NEB, M0541S). Lastly, the samples were quantified and pooled into libraries at equal molarity. Libraries were sequenced using HiSeq2500 at Tufts University Core Facility using single-end 100 bp reads. Reads were aligned to the *A. baumannii* ATCC 17978 genome and its plasmids (GenBank Accession: CP012004; pAB1: CP000522; pAB2: CP00523; pAB3: CP012005), and variants were identified at 5% cut-off using breseq 0.32^74^. Reads were aligned to *A. baumannii* ATCC 17978 reference genome (https://genomes.atcc.org/genomes/e1d18ea4273549a0) to include an additional 44 kb gene cluster^75^. The identified mutations are listed in Supplemental Dataset 2. The predicted functional impact of substitution variants was determined using PROVEAN^76^.

### Persistence assay

*A. baumannii* was grown in 3 individual LB broth cultures overnight from separate colonies. The cultures were diluted 1,000-fold into 8 ml LB broth and incubated by rotation at 37 °C for 2–3 h until reaching mid or late exponential growth (A_600nm_ ~ 0.3–0.8). Ciprofloxacin was added to reach a final concentration of 10 μg ml^−1^, the concentration regarded as 20× the MIC for the parent ATCC 17978 *A. baumannii* strain. These cultures were then incubated at 37 °C for up to 24 h. After 0, 1, 3, 6 and 24 h, 500 μl of culture was removed, washed twice in 1× PBS, resuspended in 500 μl PBS and sequentially diluted in 1× PBS. Serial dilutions were used for c.f.u. quantification by spot plating on LB agar plates. Dilution factors harbouring quantities ranging from 3–35 c.f.u. were used to calculate the c.f.u. per ml. The limit of detection by this assay was determined to be 375 c.f.u. The c.f.u. per ml across the biological replicates at each timepoint throughout the drug challenge were plotted as mean ± s.e.m.

### RT-qPCR gene expression analysis

Bacteria were grown to early stationary phase in LB broth. RNA was collected and purified using RNeasy kit (Qiagen: 74106), followed by cDNA (complementary DNA) synthesis using SuperScript VILO cDNA kit (Invitrogen, 11754050). The cDNA was then amplified with PowerUp SYBR Green Master Mix (Applied Biosystems, A25742) via a StepOnePlus Real-Time PCR system (Applied Biosystems, 4376600) following the manufacturer’s instructions, and target amplification efficiency was evaluated by generating a standard curve with dilutions of cDNA (>95% amplification efficiency for each primer pair). Primers were designed to amplify regions of around 150 bp internal to genes (Supplementary Table 2). Triple technical replicates were examined per biological sample and at least three biological replicates per strain were tested, with controls lacking reverse-transcriptase included to verify a lack of contaminant genomic DNA. Transcript levels of specific targets from each strain were evaluated by the comparative 2^-ΔΔCt^ method to the parental strain, normalizing to 2^-^^ΔΔCt^ value of the endogenous control 16s (16S ribosomal RNA). The transcript level for each target across biological replicates was plotted as mean ± s.e.m. (Figs. 3 and 4). When needed, statistical analysis was performed using two-tailed Student’s *t*-test against the mean fold change of WT using GraphPad Prism v9.

### MIC determination

MICs were determined by broth microdilution. Overnight cultures of strains of interest were diluted 1,000× in fresh LB broth and grown to mid-logarithmic phase (A_600_ ~ 0.5). Afterwards, cultures were diluted to a final A_600_ = 0.003 and tested in the presence of 2-fold dilutions of antibiotics. Culture–antibiotic mixture (200 μl) was then aliquoted to a 96-well plate (COSTAR) and technical duplicates were performed in a Biotek plate reader with rotation. Growth was monitored by measuring A_600_ at 15 min intervals for 16 h, and the MIC was determined as the lowest concentration of drug that prevented growth, using at least three biological replicates for each strain. The MICs across biological replicates were plotted as mean ± s.e.m. (Fig. 4). Statistical significance was tested using one-way ANOVA followed by Dunnett’s multiple comparison using GraphPad Prism v9.

### Molecular cloning and mutant construction

Plasmids and primers used in this study are listed in Supplementary Table 2. The mutant strains were constructed through sequential cloning into pUC18^77^ then pJB4648^78^ as previously described^13^. Briefly, the mutant allele from each strain of interest was amplified alongside upstream and downstream segments to generate a PCR product ~1,500 bp in length flanked by appropriate restriction sites. The PCR product was then ligated into pUC18 and propagated in *E. coli* DH5α. The PCR product was then subcloned from pUC18 into pJB4648 and propagated in *E. coli* DH5α λpir. After sequence confirmation, the pJB4648 plasmid construct containing the desired mutation was introduced into *A. baumannii* via electroporation. Markerless, in-frame mutations were isolated via homologous recombination as described^13^.

### Comparative sequence analysis

Genome sequences from 8,666 *A. baumannii* clinical isolates and ciprofloxacin resistance profiles for 2,618 isolates were downloaded from the PATRIC database in November 2021 as described in results. MLST analysis was performed using Pasteur scheme^39^ with the publicly available tool ‘mlst’ (https://github.com/tseemann/mlst). Of the 2618 entries, 2045 were non-repetitive and with whole genome sequences available. The LpxD protein sequence from ATCC 17978 was compared to these 2,045 isolates using ‘tblastn’ to identify the amino acid locations of mismatches and gaps. The nucleotide sequences with the best matches to the LpxD protein were extracted and translated into protein sequences. For each isolate, the translated LpxD sequence was compared to the reference LpxD sequence from AB17978. The non-synonymous changes were recorded for each clinical isolate. The non-synonymous changes at each residue were then summarized to reflect the total number of clinical isolates showing those changes. The raw numbers were further transformed into *z*-scores to determine whether a particular residue was significantly overrepresented by non-synonymous changes linked to CIP^R^ or CIP^S^ phenotypes (Extended Data Fig. 1). To reduce clonal effects, one clinical isolate per ST group was selected randomly. Multiple aligned LpxD protein sequences are shown in Supplementary Dataset 3. One isolate had no hit for LpxD. The MLST profiles from 7 housekeeping genes were used to calculate the Euclidean distance, and hierarchical clustering was determined using R (Fig. 5a). A phylogenetic tree from these 139 genomes was built using mashtree^79^ and visualized in Geneious Prime (https://www.geneious.com/) (Extended Data Fig. 3). The presence and absence of isolates were summarized and visualized in a heatmap using GraphPad Prism v9 (Fig. 5b and Extended Data Fig. 2). Within each group, the mismatches/gaps at each amino acid location within LpxD were displayed as number of genomes having mismatches/gaps at that location. A table of the data was constructed showing the specific amino acid in the rows, the genome groups harbouring these mutations in the columns, and the total number of mismatches/gaps in the cells. The results in each cell were then normalized using *z*-scores with the mean and standard deviation from individual genome groups. The *z*-score was plotted as function of each residue.

### Calculation of doubling time in broth culture

Overnight cultures of bacteria were diluted 1,000-fold in fresh LB broth and grown for 8 h. The A_600_ values were measured every hour using a spectrophotometer, and the log_2_-transformed values were plotted as a function of time (h). To calculate the doubling time, a modified sigmoid function $\left( {y = \frac{{L1}}{{e^{ - k(x - x_{0})} + 1}} - L2} \right)$ was fitted to the plotted curve (*y*, log_2_-transformed A_600_ value; *x*, time in h; *x*_0_, timepoint when the growth rate was the fastest; *k*, *L*1, *L*2 are rate constants). The fastest growth rate for each culture was determined as the time in which the second derivative of the fitted curve approached 0 (or when *x* *=* *x*_0_), at which point the doubling time was represented as the inverse of the slope (∆*t* = $4/(k \times L1)$) (code available at: https://github.com/huoww07/calulate_bacteria_doubling_time). To evaluate the goodness of fit, the *R*^2^ value was calculated for each growth curve assay (the *R*^2^ ranges from 0 to 1, representing the worst to the best fit). At least three biological replicates were performed for each test strain (Supplementary Tables 3 and 4) and the average doubling time and standard deviation were calculated. Statistical significance was determined using one-way ANOVA followed by Dunnett’s multiple comparisons in R 4.1.2.

### Statistics, software and visualization

Data analysis and statistics were performed in GraphPad Prism 9 or R 4.1.2, using packages including tidyverse, knitr and DescTools. Data visualization was performed using GraphPad Prism 9.

### Reporting summary

Further information on research design is available in the Nature Research Reporting Summary linked to this article.

## Supplementary information

Supplementary Information Supplementary Tables 1–4.

Reporting Summary

Supplementary Data 1 Raw MLST results.

Supplementary Data 2 Mutation frequencies for reported isolates.

Supplementary Data 3 LpxD sequences from 139 clinical isolates having different MLST profiles.

## Data Availability

Sequencing reads that support the findings of this study (Fig. 2) are deposited in the NCBI SRA with the accession code PRJNA485355. Detailed accession numbers for each sample are listed in Supplementary Table 1. Sequencing reads were analysed by breseq (https://github.com/barricklab/breseq) and all variants can be found at https://github.com/huoww07/Ab_evolutionary_pathways. Variants were filtered against parental WT AB17978 and results are attached as Supplementary Dataset 2. *A. baumannii* genome data used in this study (Fig. 5) are available in the PATRIC database (patricbrc.org) with the sequence IDs listed in Supplementary Dataset 1. Source data are provided with this paper.

## Source data

Source Data Fig. 1
Source Data Fig. 2
Source Data Fig. 3
Source Data Fig. 4
Source Data Fig. 5
Source Data Fig. 6
Source Data Extended Data Fig. 1
Source Data Extended Data Fig. 2
Source Data Extended Data Fig. 4

## Code availability

All tools are publicly available. MLST analysis was performed using Pasteur scheme with the publicly available tool ‘mlst’ (https://github.com/tseemann/mlst). A phylogenetic tree from select 139 genomes was built using mashtree (https://github.com/lskatz/mashtree). Bacterial doubling time was computed using customized Python scripts available at https://github.com/huoww07/calulate_bacteria_doubling_time.
