# Supplementary material for: Immunosuppression broadens evolutionary pathways to drug resistance and treatment failure during Acinetobacter baumannii pneumonia in mice
Source: Nat Microbiol. 2022 May 26;7(6):796–809. doi: 10.1038/s41564-022-01126-8 (PMC9159950; doi:10.1038/s41564-022-01126-8)
Supplement: Supplementary file 1 — Supplementary Tables 1–4. [file 41564_2022_1126_MOESM1_ESM.pdf]

nature portfolio

https://doi.org/10.1038/s41564-022-01126-8

Supplementary information

Immunosuppression broadens evolutionary pathways to drug resistance and treatment failure during *Acinetobacter baumannii* pneumonia in mice

In the format provided by the authors and unedited

Table S1. Strains used in this study.

Clone name	Description	Accession	Cipro MIC	reference	Relevant alleles
DH5α	*E. coli:* F$^-$ *endA1 glnV44 thi-1 recA1 relA1 gyrA96 deoR nupG purB20* φ80d*lacZ*ΔM15 Δ(*lacZYA-argF*)U169, hsdR17($r_K^- m_K^+$), λ$^-$	N/A	N/A	70	N/A
DH5α λ*pir*	DH5α encoding π protein	N/A	N/A	71	N/A
WT/AB17978	*A. baumannii* ATCC 17978	SRX9736139	0.5	72	N/A
Ab12	Single colonies selected without CIP from immunocompetent L1: 15 passages	SRX9736138	0.5	This study	*ispB* R49H; ΔCP012005
Ab11		SRX12722651	0.5	This study	*ispB* R49H; *acx60_02425* V138I
Ab21	Single colonies selected without CIP from immunocompetent L2: 15 passages	SRX9736117	0.5	This study	*ispB* R49H; soxF V374*fs*
Ab22		SRX9736106	0.5	This study	*adeS* F114L; ACX60_04790 S27fs
Ab31	Single colonies selected without CIP from immunocompetent L3: 15 passages	SRX9736104	0.5	This study	*alaA* H391fs
Ab33		SRX12722652	0.5	This study	MrcB 768delN
CIPP10 Ab11	Single colonies CIP 1 µg/mL selection from immunocompetent L1: 10 passages	SRX12722636	4	This study	*obg* Δ12bp; AdeL C312*; Mmg V193M; PntB P363S
CIPP10 Ab12		SRX12722647	2	This study	AdeL C312*; Mmg V193M; PntB P363S
CIPP10 Ab13		SRX12722621	2	This study	AdeL C312*; Mmg V193M; PntB P363S
CIPP10 Ab14		SRX12722622	2	This study	AdeL C312*; RsgA Q45*
CIPP10 Ab21	Single colonies CIP 1 µg/mL selection from immunocompetent L2: 10 passages	SRX12722623	1	This study	AdeL C312*
CIPP10 Ab22		SRX12722624	1	This study	AdeL C312*
CIPP10 Ab23		SRX12722625	1	This study	AdeL C312*; GyrA R241C
CIPP10 Ab24		SRX12722626	1	This study	AdeL C312*; $utcC_{intergenic}$

CIPP10 Ab31	Single colonies CIP 1 μg/mL selection from immunocompetent L3: 10 passages	SRX12722653	1	This study	AdeL C312*; ComL K197N; ΔCP012005
CIPP10 Ab32		SRX12722627	1	This study	AdeL C312*; ComL K197N
CIPP10 Ab33		SRX12722628	1	This study	AdeL C312*; ComL K197N
CIPP10 Ab34		SRX12722629	1	This study	AdeL C312*; ComL K197N
CIPP15 Ab21	Single colonies CIP 1 μg/mL selection from immunocompetent L2: 15 passages	SRX12722630	1	This study	RplN M7L
CIPP15 Ab22		SRX12722631	1	This study	AdeL C312*
CIPP15 Ab23		SRX12722632	1	This study	PrmA S217I; ACX60_07895 A43fs
CIPP15 Ab24		SRX12722633	1	This study	AdeL C312*
CIPP15 Ab25		SRX12722634	1	This study	AdeL C312*
CIPP15 Ab26		SRX12722635	1	This study	AdeL C312*
Cy11	Single colonies selected without CIP from neutrophil-depleted mice lineage 1: 15 passages	SRX9736134	4	This study	g*yrB* Q447H; *adeL* C312*
Cy12		SRX9736133	4	This study	*gyrB* Q447H; *adeL* C312*
Cy21	Single colonies selected without CIP from neutrophil-depleted mice lineage 2: 15 passages	SRX9736132	8	This study	*gyrA* A117E
Cy22		SRX9736131	8	This study	*gyrA* A117E
Cy31	Single colonies selected without CIP from neutrophil-depleted mice lineage 3: 15 passages	SRX9736130	4	This study	*acfR* V136D; *adeL* P131H; *lpxD* insA; *mfsA*(-9)
Cy32		SRX9736129	4	This study	*acfR* V136D; *adeL* P131H; *lpxD* insA; *mfsA*(-9)
CIPCy11	Single colonies selected with CIP 1 μg/mL from neutrophil-depleted mice lineage 1: 15 passages	SRX12722637	4	This study	*gyrB* Q447H; *adeL* C312*
CIPCy13		SRX12722638	4	This study	*gyrB* Q447H; *adeL* C312*

CIPCy21	Single colonies selected with CIP 1 μg/mL from neutrophil-depleted mice lineage 2: 15 passages	SRX12722639	4	This study	*acfR* V136D; *adeL* P131H; *lpxD* insA; *mfsA*(-9)
CIPCy22		SRX12722640	4	This study	*acfR* V136D; *adeL* P131H; *lpxD* insA; *mfsA*(-9)
CIPCy32	Single colonies selected with CIP 1 μg/mL from neutrophil-depleted mice lineage 3: 15 passages	SRX12722641	4	This study	*acfR* V136D; *adeL* P131H; *lpxD* insA; *mfsA*(-9)
CIPCy33		SRX12722642	4	This study	*acfR* V136D; *adeL* P131H; *lpxD* insA; *mfsA*(-9)
P9Cy2.1	Single colony selected from neutrophil-depleted mice lineage 2: 9 passages	SRX9736110	1	This study	*adeL* Y324*
P9Cy3.1	Single colony selected from neutrophil-depleted mice lineage 3: 9 passages	SRX9736109	1	This study	*adeL* ΔI335A336
WH_B3	AB17978 *gyrB* Q447H	SRX9736122	0.5	This study	*gyrB* Q447H
WH113	AB17978 *adeL* C312*	SRX9736121	1	This study	*adeL* C312*
WH114	AB17978 *lpxD* T118_A119insA	SRX9736120	0.25	This study	*lpxD* T118_A119insA
WH309	AB17978 *lpxD* E117K	SRX9736119	0.25	This study	*lpxD* E117K
WH115	AB17978 *acfR* V136D	SRX9736118	0.25	This study	*acfR* V136D
WH_mfsA_48	AB17978 *mfsA*(-9)	SRX9736116	0.75	This study	*mfsA*(-9) A->G
WH_mfsA_12	AB17978 *mfsA*(-19)	SRX9736115	0.75	This study	*mfsA*(-19) T->A
WH319	AB17978 *mfs*(-9) *adeL* P131H	SRX9736114	4	This study	*mfs*(-9); *adeL* P131H
WH316	AB17978 *mfs*(-19) *adeL* P131H	SRX9736113	2	This study	*mfs*(-19); *adeL* P131H
JH1	AB17978 *mfsA*(-19) *adeL* ΔI335A336	SRX9736112	3	This study	*mfsA*(-19); *adeL* ΔI335A336
WH124	AB17978 *lpxD* insA *acfR* V136D *adeL* P131H	SRX9736111	0.75	This study	*lpxD* insA; *acfR* V136D; *adeL* P131H
EHA8	AB17978 *adeL* ΔIA	SRX9736108	1	This study	*adeL* ΔI335A336
EHA15	AB17978 *adeL* P131H	SRX9736107	1	This study	*adeL* P131H
EHA123	AB17978 *adeL* ΔIA Δ*adeFGH*	SRX9736105	1	This study	*adeL* ΔI335A336; Δ*adeFGH*

Table S2. Plasmids and primers used in this study.

Plasmid	Description	Reference
pUC18	*oriColE1* MCS AmpR	68
pJB4648	R6K ori (E. coli), GentR	69,71
**Primers**	**Sequence**	
**qRT_PCR primers**		
16s_qRT_For	CAGCTCGTGTCGTGAGATGT	
16s_qRT_Rev	CGTAAGGGCCATGATGACTT	
adeG_qRT_For	tgctgaaacggttgcatctc	
adeG_qRT_Rev	acggttttgaaccaattgttgg	
adeB_qRT_For	tccggtacagcagagattac	
adeB_qRT_Rev	tgaagcctcaacctgtaaac	
adeJ_qRT_For	cttgaagaacgtgagtctgg	
adeJ_qRT_Rev	attgcaccaatgacaccaag	
15145_qRT_For	GGGCACATACGACGAACTGA	
15145_qRT_Rev	CAAGCGCCACATTTTGACCA	
15150_qRT_For	GTTTTCCGCCAACTCAGCAC	
15150_qRT_Rev	ATCGCCAACGCAAATAGGGA	
**Mutant construct primers**		
mfsA_For_BamHI	CGCGGATCCTGTTTAACCTGCAACTGCGC	
mfsA_Rev_SalI	ACGCGTCGACCAAGCCTTCAGGACCGACTT	
AdeL_For_BamHI	CGCGGATCCGAAATGTACGGCAATGAG	
AdeL_Rev_Long_SalI	ACGCGTCGACCGAATCACCTTCCAATCCTT	
AraC_For_NotI	ATAAGAATGCGGCCGCCCGTGTTGAGCTTCTTGTTT	
AraC_Rev_XmaI	TCCCCCCGGGCGTCTACTCCTGCCGCAA	
LpxD_For_BamHI	CGCGGATCCCTGGTTATGGGGTAATTGATC	
LpxD_Rev_SalI	ACGCGTCGACGGTTTTGGCGTTTCATTGT	
GyrB_For_BamHI	CGCGGATCCGCCTCTCAAACAGAACAAACC	

GyrB_Rev_SalI	ACGCGTCGACGTGGTTCAACATCATCGCCC

Table S3. *in vitro* growth rates of LpxD mutants compared to AB17978.

Time (hr)		AB17978				AB17978::LpxD T118_A119insA				AB17978::LpxD E117K			
Optical density at 600nm	0	0.003	0.004	0.005	0.004	0.003	0.004	0.004	0.004	0.004	0.004	0.004	0.004
	1	0.015	0.015	0.015	0.013	0.009	0.011	0.011	0.011	0.014	0.014	0.014	0.013
	2	0.144	0.138	0.143	0.131	0.054	0.059	0.064	0.067	0.124	0.132	0.129	0.12
	3	1.085	1.01	1.01	0.97	0.335	0.37	0.395	0.43	0.935	0.975	0.985	0.825
	4	2.47	2.68	2.445	2.38	1.16	1.27	1.355	1.39	2.36	2.475	2.475	2.27
	5	3.79	3.82	3.78	3.8	2.81	2.98	2.92	3.12	3.64	3.79	3.64	3.76
	6	4.26	4.23	4.3	4.29	3.56	3.52	3.57	3.81	4.41	4.31	4.33	4.12
	7	4.5	4.59	4.67	4.64	3.95	3.97	3.99	4.09	4.69	4.4	4.47	4.55
	8	4.3	4.29	4.58	4.64	4.34	4.36	4.23	4.28	4.44	4.44	4.49	4.32
**Doubling time(min)**		**17.325**	**17.479**	**17.667**	**17.258**	**21.579**	**21.904**	**21.563**	**21.165**	**17.927**	**17.511**	**17.546**	**18.005**
**R^2**		**0.999**	**1**	**0.995**	**0.996**	**1**	**1**	**1**	**0.999**	**0.999**	**0.999**	**0.999**	**0.998**

Table S4. *in vitro* growth rates of the isolates acquired from the *in vivo* passaging experiments.

**Time (hr)**		**AB17978**			**AB17978::*adeL* ΔIA**			**Cy11**			**Cy21**			**Cy31**		
Optical density at 600nm	0	0.001	0.001	0.001	0.001	0.001	0.001	0.001	0.001	0.001	0.001	0.001	0.001	0.001	0.001	0.001
	1	0.004	0.002	0.004	0.003	0.003	0.003	0.003	0.003	0.003	0.003	0.003	0.003	0.003	0.004	0.003
	2	0.02	0.021	0.025	0.013	0.015	0.014	0.012	0.011	0.011	0.014	0.012	0.013	0.017	0.015	0.015
	3	0.106	0.182	0.201	0.074	0.077	0.055	0.034	0.042	0.03	0.059	0.042	0.049	0.109	0.052	0.094
	4	0.566	0.712	0.747	0.417	0.416	0.284	0.111	0.235	0.084	0.33	0.22	0.222	0.532	0.272	0.488
	5	1.6	1.65	1.8	1.22	1.2	0.99	0.65	0.9	0.51	1.02	0.93	1.95	1.23	1.03	1.24
	6	2.03	2.04	2.12	1.44	1.38	1.3	1.06	1.23	0.94	1.35	1.23	1.22	1.55	1.36	1.55
	7	2.3	2.29	2.5	1.69	1.76	1.63	1.38	1.54	1.1	1.57	1.5	1.39	1.88	1.74	1.78
	8	2.56	2.55	2.7	1.85	1.83	1.89	1.51	1.73	1.37	1.78	1.61	1.57	2.01	1.91	2
**Doubling time(min)**		**21.463**	**17.147**	**19.693**	**20.828**	**21.57**	**23.843**	**27.629**	**23.331**	**28.985**	**22.828**	**23.691**	**21.038**	**20.606**	**24.404**	**20.682**
**R^2**		**0.993**	**0.986**	**0.999**	**0.996**	**0.997**	**0.996**	**0.987**	**0.992**	**0.984**	**0.995**	**0.99**	**0.975**	**0.999**	**0.984**	**0.998**
