# Supplementary material for: Immunosuppression broadens evolutionary pathways to drug resistance and treatment failure during Acinetobacter baumannii pneumonia in mice
Source: Nat Microbiol. 2022 May 26;7(6):796–809. doi: 10.1038/s41564-022-01126-8 (PMC9159950; doi:10.1038/s41564-022-01126-8)
Supplement: Supplementary file 2 — Reporting Summary [file 41564_2022_1126_MOESM2_ESM.pdf]

nature portfolio

Corresponding author(s): Ralph Isberg

Last updated by author(s): Mar 23, 2022

Reporting Summary

Nature Portfolio wishes to improve the reproducibility of the work that we publish. This form provides structure for consistency and transparency in reporting. For further information on Nature Portfolio policies, see our Editorial Policies and the Editorial Policy Checklist.

Statistics

For all statistical analyses, confirm that the following items are present in the figure legend, table legend, main text, or Methods section.

n/a	Confirmed	
☐	☒	The exact sample size (*n*) for each experimental group/condition, given as a discrete number and unit of measurement
☐	☒	A statement on whether measurements were taken from distinct samples or whether the same sample was measured repeatedly
☐	☒	The statistical test(s) used AND whether they are one- or two-sided<br>*Only common tests should be described solely by name; describe more complex techniques in the Methods section.*
☐	☒	A description of all covariates tested
☐	☒	A description of any assumptions or corrections, such as tests of normality and adjustment for multiple comparisons
☐	☒	A full description of the statistical parameters including central tendency (e.g. means) or other basic estimates (e.g. regression coefficient) AND variation (e.g. standard deviation) or associated estimates of uncertainty (e.g. confidence intervals)
☐	☒	For null hypothesis testing, the test statistic (e.g. *F*, *t*, *r*) with confidence intervals, effect sizes, degrees of freedom and *P* value noted<br>*Give P values as exact values whenever suitable.*
☒	☐	For Bayesian analysis, information on the choice of priors and Markov chain Monte Carlo settings
☒	☐	For hierarchical and complex designs, identification of the appropriate level for tests and full reporting of outcomes
☒	☐	Estimates of effect sizes (e.g. Cohen's *d*, Pearson's *r*), indicating how they were calculated

Our web collection on statistics for biologists contains articles on many of the points above.

Software and code

Policy information about availability of computer code

Data collection	No software was used.
Data analysis	Code availability statement is added in the manuscript. All tools are publicly available. MLST analysis was performed using Pasteur scheme with the publicly available tool “mlst” (https://github.com/tseemann/mlst). A phylogenetic tree from select 139 genomes was built using mashtree (https://github.com/lskatz/mashtree). Bacterial doubling time was computed using customized python scripts: https://github.com/huoww07/calulate_bacteria_doubling_time. Data analysis and statistics were performed in Prism GraphPad 9 or R 4.1.2, using packages including tidyverse, knitr and DescTools. Data visualization was performed using Prism GraphPad version 9.

For manuscripts utilizing custom algorithms or software that are central to the research but not yet described in published literature, software must be made available to editors and reviewers. We strongly encourage code deposition in a community repository (e.g. GitHub). See the Nature Portfolio guidelines for submitting code & software for further information.

Data

Policy information about availability of data

All manuscripts must include a data availability statement. This statement should provide the following information, where applicable:

- Accession codes, unique identifiers, or web links for publicly available datasets
- A description of any restrictions on data availability
- For clinical datasets or third party data, please ensure that the statement adheres to our policy

Data availability statement is added in the manuscript. Sequencing reads that support the findings of this study (Fig 2) are deposited in NCBI SRA with the accession code PRJNA485355. Detailed accession numbers for each sample are listed in Supplementary Table S1. Sequencing reads were analyzed by breseq (https://github.com/barricklab/breseq) and all variants can be found at https://github.com/huoww07/Ab_evolutionary_pathways. Variants were filtered against parent al

WT AB17978 and results are attached as Supp. Dataset 2. A. baumannii genome data used in this study (Fig 5) are available in the PATRIC database (patricbrc.org) with the sequence IDs listed in Supp. Dataset 1.

Field-specific reporting

Please select the one below that is the best fit for your research. If you are not sure, read the appropriate sections before making your selection.

☒ Life sciences ☐ Behavioural & social sciences ☐ Ecological, evolutionary & environmental sciences

For a reference copy of the document with all sections, see nature.com/documents/nr-reporting-summary-flat.pdf

Life sciences study design

All studies must disclose on these points even when the disclosure is negative.

Sample size	Sample size of 3 was selected for animal experiments to minimize the use of animals. For confirmation using single colonies, 2 single colonies were selected under each condition, resulting in sample size of 6 for each condition. Reproducibility and consistency was observed among 6 samples, confirming the sufficiency. All mice are housed in ventilated caging systems (10-15 air changes/hour), at temperatures of 68-79°F (~20-26°C) and 30-70% humidity with 12-hr light/12-hr dark cycle.
Data exclusions	No data was excluded.
Replication	The reproducibility of evolution experiment was assessed using three mice in parallel for each procedure. The results showed different trajectories, which was expected due to the nature of evolution. For all other experiments, the reproducibility was assessed by repeating the same experiment using at least three biological replicates total. High level of reproducibility was indicated by small standard error of mean (SEM) or standard deviation (SD).
Randomization	All animals in the study are 6-8 weeks old BALB/C mice. Neutrophil depletion procedure was randomly performed on mice. Use of immunocompromised and neutrophil depleted mice during all infection procedures were randomly selected. Selection of single colonies was performed randomly, with similar size and morphology.
Blinding	Use of immunocompromised animals cannot be blinded as they are readily identified by their increased lethargy and disease susceptibility relative to untreated animals. In experiments in which we are not comparing immunocompromised mice (Fig. 6), Fig 6A,B involve mice that are technical replicates or biological replicates, so there are no comparison mice, all the infections are identical with identical strains and identical mice. For 6C, blinding of mice is not necessary because we are phenotyping bacteria. We cannot blind the plates, because the different strains require different plating conditions, so the experiment would be impossible to blind, or would require using at minimum 6 times the amount of media to properly blindMIC determinations are not blinded but are highly reproducible as clearly demonstrated by individual data points shown in the figures. Furthermore, each well is time and date stamped using the acquisition software, so the data are accumulated on top of a time and date stamp that prevents intentional sample switching.

Reporting for specific materials, systems and methods

We require information from authors about some types of materials, experimental systems and methods used in many studies. Here, indicate whether each material, system or method listed is relevant to your study. If you are not sure if a list item applies to your research, read the appropriate section before selecting a response.

Materials & experimental systems

n/a	Involved in the study	
☒	☐	Antibodies
☒	☐	Eukaryotic cell lines
☒	☐	Palaeontology and archaeology
☐	☒	Animals and other organisms
☒	☐	Human research participants
☒	☐	Clinical data
☒	☐	Dual use research of concern

Methods

n/a	Involved in the study	
☒	☐	ChIP-seq
☒	☐	Flow cytometry
☒	☐	MRI-based neuroimaging

Animals and other organisms

Policy information about studies involving animals; ARRIVE guidelines recommended for reporting animal research

Laboratory animals	6-8 weeks-old female BALB/C mice. 12 light/12 dark cycle. Temperatures of 65-75°F (~18-23°C) with 40-60% humidity.
Wild animals	no wild animals were used in the study.
Field-collected samples	no field collected samples were used in the study.

Ethics oversight	All animal procedures were approved by the Institutional Animal Care and Use Committee (IACUC) of Tufts University. The animal care and use program at Tufts University/Tufts Medical Center – Boston Campus has been continuously accredited by AAALAC since 18 April 1966 with the most recent date of March 21, 2014. The program is inspected by the USDA under the research license 14-R-0082 and holds the Public Health Service Policy Assurance number A3775-01.

Note that full information on the approval of the study protocol must also be provided in the manuscript.
